# Strong, Fast-Response Printable Lignin/PNIPAM Thermo-Responsive Hydrogel via Hierarchical Phase Separation

**DOI:** 10.3390/gels12050362

**Published:** 2026-04-27

**Authors:** Qian Wang, Huijie Zhang, Wenlong Zhang, Linbin Li, Yifan Zhang, Ping Rao, Xiangyu You

**Affiliations:** 1 College of Bioresources Chemical and Materials Engineering, Shaanxi University of Science &Technology, Xi’an 710021, China; 18791877277@163.com (Q.W.); zhangwenlong0803@126.com (W.Z.); 18691608697@163.com (L.L.); xyyou@sust.edu.cn (X.Y.); 2 Department of Engineering Mechanics, Zhejiang University, Hangzhou 310027, China; 12424116@zju.edu.cn; 3 State Key Laboratory of Fluid Power & Mechatronic System, Center for X-Mechanics, Hangzhou 310027, China; 4 Key Laboratory of Soft Machines and Smart Devices of Zhejiang Province, Center for X-Mechanics, Hangzhou 310027, China

**Keywords:** hydrogel, thermo-responsive hydrogel, high-volume shrinkage, lignin, 4D print

## Abstract

Stimuli-responsive hydrogels have gained significant attention as one of the most attractive materials for soft robots. Herein, a facile, printable thermo-responsive hydrogel (NL hydrogel) with rapid volume change capability and excellent mechanical properties was developed through the self-assembly of poly(N-isopropylacrylamide) (PNIPAM) and hydrophobic lignin. The lignin and PNIPAM self-assembled into a hierarchical phase-separated structure consisting of lignin-rich dense regions with a bicontinuous morphology and PNIPAM-rich, chain-sparse regions. This unique architecture results in multiscale water channels, enabling an ultrafast dehydration response (expelling 90% of its water within 10 s) and an ultrahigh volume shrinkage of up to 96.4% above its lower critical solution temperature (LCST). The phase separation structure also endows the NL hydrogels with outstanding mechanical properties, achieving tensile stress and strain values exceeding 1 MPa and 500% below the LCST, and approximately 5 MPa and 1500% above the LCST. The responsive speed and mechanical properties of the NL hydrogels surpass those of most reported thermo-responsive hydrogels. The NL hydrogels can be readily printed via direct ink writing into various geometries. The printed NL hydrogels demonstrate thermo-triggered shape morphing, functioning as temperature-controlled actuators with adjustable curvature and as manipulators for capture, wrapping, encapsulation, and switching. Furthermore, the photothermal effect of lignin enables light-controlled actuation of the NL hydrogel.

## 1. Introduction

Soft robots [1,2,3,4,5] have garnered significant research interest due to their exceptional deformability, enabling operation in complex environments where conventional rigid robots face limitations [6]. Among the potential materials, stimuli-responsive hydrogels [7], capable of reacting to environmental stimuli such as temperature, pH, and light, are a particularly promising owning to their porous structure and significant volume change capabilities [8,9,10,11,12,13,14]. By assembling bilayers of stimuli-responsive hydrogel with different volume-changing ratios [15], stimuli sensitivity [16], or mechanical properties [17], or by generating asymmetrical structures [18], stimuli-responsive hydrogels with bending/unbending capabilities for a soft actuator and manipulator can be created.

Compared to other stimuli, temperature variation is easily realized and controlled, making thermo-responsive hydrogels particularly advantageous for practical applications [19,20,21,22,23,24,25]. For instance, utilizing the thermo-responsive VSNPs-P(NIPAM-co-AA) hydrogel, composed of vinyl functionalized silica nanoparticles and poly(N-isopropylacrylamide-co-acrylic acid), had large volume shrinking ratios of up to 72.5%. This hydrogel shows potential for applications in intelligent soft actuators and artificial robots [15]. Kim et al. [26] employed UV-crosslinking technology to fabricate a programmable actuation system through gradient crosslinking of thermo-responsive hydrogels, achieving precise deformation control via LCST regulation.

Despite these advancements, thermo-responsive hydrogels still face critical limitations. The inefficient water transport channels often result in low volume change ratios (typically below 50%) and slow response rates, hindering real-time control. To address these issues, three primary strategies have been developed to construct efficient water transport channels: (1) creating highly porous networks [27,28,29,30]; (2) utilizing the fast shrinkage of thermo-responsive microgels embedded in the bulk thermo-responsive hydrogel to create water channels to enhance water expulsion [31]; and (3) using nanogels as a crosslinker to generate multi-scaled pores as water pass channels [15,32]. While these strategies have enabled high volume change ratios (72.5–97%) and highly shortened equilibrium times (1–20 min) [15,27,28,29,30,31,32], a fundamental trade-off remains between mechanical strength and response kinetics. Generally, increasing the mechanical strength of a hydrogel requires a higher molecular chain density or crosslinking degree, which inevitably constricts the water transport channels and hinders rapid water flow. As a result, most existing thermo-responsive hydrogels with high volume change ratios are mechanically weak, with the tensile stress barely exceeding 1 MPa [15,33,34,35,36,37,38,39,40], which is insufficient to generate a high actuation force for high-load soft crawlers and grippers. Furthermore, the complex polymerization and crosslinking processes required for these specialized architectures often limit their compatibility with advanced fabrication techniques like 4D printing.

Lignin is the second most abundant biopolymer after cellulose, and the primary aromatic resource in plants. Currently, global lignin production reaches approximately 100 million tonnes annually, primarily generated as a byproduct from the pulp and paper industry and second-generation bioethanol production. Despite this vast availability, lignin remains significantly underutilized; approximately 98% of industrial lignin is currently burnt as low-value fuel to recover energy, and the high-value valorization of lignin is urgently required to unlock its full potential [41]. In recent years, the strategic integration of lignin has been shown to impart hydrogels with exceptional properties beyond simple fillers. For instance, Ni et al. developed an ultrastrong lignosulfonate (LS)/polyvinyl alcohol (PVA) hydrogel by leveraging LS-induced crystallization [42]. Building on the self-aggregation of hydrophobic lignin, our previous studies demonstrated that a facile solvent drying-water swelling process could produce lignin-based hydrogels with superior stiffness and toughness [43,44].

Additionally, 4D printing of natural materials has gained increasing interest for sustainable smart structures. While hydrophilic polysaccharides and proteins are widely explored, their hydrophilic nature and chain entanglements often result in a slow actuation time (sometimes exceeding 1 h) of the smart materials triggered by hydration/dehydration and/or temperature [45]. Lignin’s rigidity and hydrophobicity offer a unique chance to bridge this gap for high-performance 4D actuation.

**Figure 1:**
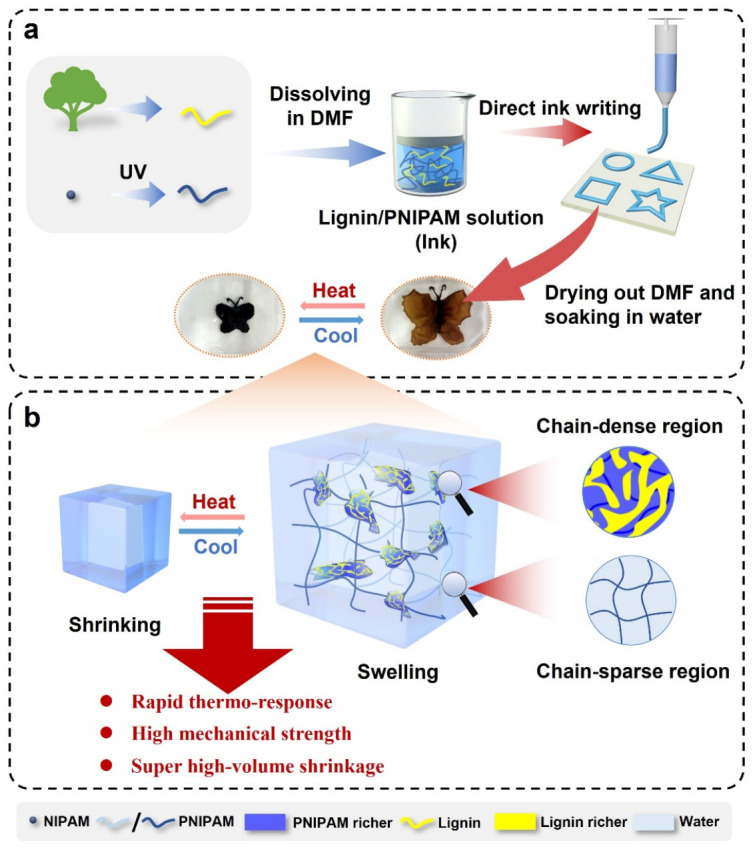
(**a**) Schematic illustration of the fabrication and printing process of the PNIPAM/lignin hydrogel; (**b**) schematic representation of the hierarchical phase separation structure of the PNIPAM/lignin hydrogel with a large volume shrinkage ratio.

Building on the principle of the self-aggregation of hydrophobic lignin, herein we report a novel, printable, lignin-based thermo-responsive hydrogel (NL hydrogel) that exhibits a rapid thermal response, high volume shrinkage, high mechanical properties, and excellent actuation performance. The hydrogel was synthesized from a PNIPAM/lignin solution via a facile drying-swelling process, which can be used to print into various patterns with the direct ink writing (DIW) method (Figure 1a). The formed NL hydrogel consists of lignin-rich, chain-dense regions with a bicontinuous phase-separated structure to crosslink the PNIPAM-rich, chain-sparse regions. This architecture provides a dual-functional mechanism: the chain-dense regions provide robust mechanical reinforcement, while simultaneously creating multi-scale water transport channels alongside the chain-sparse regions (Figure 1b). Thus, NL hydrogel holds great promise for significant applications in soft robotics and sustainable smart structures.

## 2. Results and Discussion

### 2.1. Fabrication of Lignin Hydrogels

**Figure 2:**
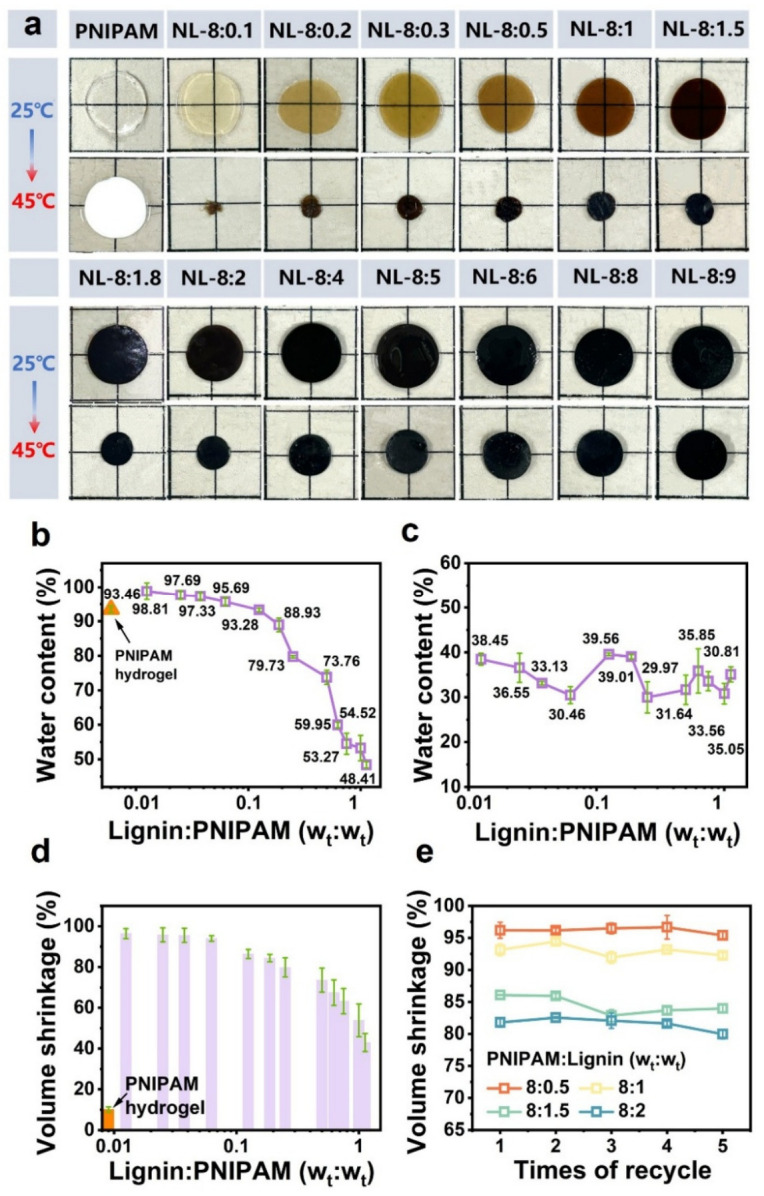
(**a**) Photos of the NL hydrogels with different lignin contents and the PNIPAM hydrogel at 25 °C and 45 °C. The water content of the PNIPAM hydrogel and the NL hydrogels vs. lignin/PNIPAM ratio: (**b**) at 25 °C and (**c**) at 45 °C. (**d**) Volume shrinkage of the NL hydrogels and PNIPAM hydrogel after heating. (**e**) Volume shrinkage of different NL hydrogels after dehydrating–swelling cycles.

PNIPAM/lignin thermo-responsive hydrogels (NL hydrogel) were fabricated via a solvent drying and swelling process (Figure 1a) without any chemical crosslinker, similar to the previously reported PDMA/lignin hydrogel [44]. During the solvent drying process, the evaporation of DMF increases the polymer concentration and promotes the chain aggregation of hydrophilic PNIPAM and hydrophobic lignin to minimize the system’s free energy. Meanwhile, the interchain entanglements between the two polymers restrict long-range chain diffusion, leading to kinetically trapped incomplete phase separation. Owing to the inherent hydrophobicity, lignin aggregates are resistant to water disruption and can interlock with PNIPAM chains to serve as physical crosslinking sites, which effectively suppress the dissolution of PNIPAM in aqueous media. It is worth mentioning that the drying process is essential for the formation of NL hydrogels, and hydrogels cannot form by directly mixing lignin and PNIPAM in water (Figure S1). The NL hydrogels were obtained by soaking the dry PNIPAM/lignin sheets in water. The photos of the equilibrium-swollen NL hydrogels with different PNIPAM to lignin ratios at the temperature below and above the LCST of PNIPAM in comparison with the covalently crosslinked PNIPAM hydrogel are shown in Figure 2a. The covalently crosslinked PNIPAM hydrogel exhibited high transparency at room temperature. Due to the limited water transport channels, a large volume of water cannot be expelled from the gel. Consequently, the PNIPAM hydrogel transitioned to an opaque state without exhibiting a noticeable volume change upon heating. The NL-8:0.1 gel, with a mass ratio of PNIPAM to lignin of 8:0.1, was transparent with a light-yellow color and had a jelly-like consistency at room temperature. With the increase in lignin content, the hydrogel gradually turned into a rigid gel with a dark-brown color. Unlike the PNIPAM hydrogel, the NL hydrogels, particularly those with a low lignin content, exhibited significant volume shrinkage after heating (Figure 1a). Notably, the jelly-like NL-8:0.1 gel, with a diameter of 13 mm at 25 °C, shrank to a tiny, plastic-like sheet with a diameter of 4.8 mm above 45 °C, corresponding to a super-high volume shrinkage value of 96.4%. The water content below (25 °C) and above (45 °C) the LCST (32 °C), as well as the volume shrinkage of the hydrogels after heating, were further measured. As shown in Figure 2b, the equilibrium water content of the swollen NL hydrogels at room temperature exhibited a progressive decrease with the increase in the lignin/PNIPAM ratio. After the shrinkage, all NL hydrogels exhibited a low water content of approximately 35 wt%, which was almost independent of the lignin content (Figure 2c). The volume shrinkage of the NL hydrogels after heating also decreased with the increase in the lignin/PNIPAM ratio (Figure 2d). Thus, the volume change ratio of the NL hydrogel can be easily tuned by adjusting the lignin content. In addition, the NL-8:9 gel, with a lignin/PNIPAM ratio higher than 1 and a low water content of ~50 wt% at room temperature, still exhibited a high volume shrinkage ratio of around 40%. In contrast, the PNIPAM hydrogel, despite its high water content of 93 wt%, only showed a volume shrinkage value of approximately 10% upon heating (Figure 2d). The high volume shrinkage of the NL hydrogels was reversible. As shown in Figure 2e, the volume shrinkage ratio of the four NL hydrogels remained almost unchanged after five dehydration–swelling cycles, indicating the structural stability of the NL hydrogels.

**Figure 3:**
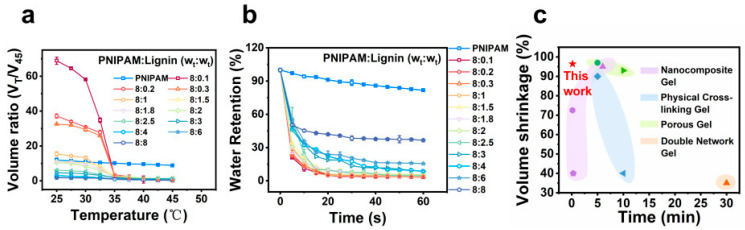
(**a**) The volume ratio of NL hydrogels vs. temperature from 25 to 45 °C. (**b**) Water retention of NL hydrogels vs. soaking time in water at 45 °C. (**c**) Comparison of volume shrinkage and thermo-response time of NL hydrogels in this study with the thermo-responsive hydrogels in other reported studies [15,27,32,33,34,35,36,37,38,39,40,47,48,49,50].

In addition to the significant and tunable volume change ratios, NL hydrogels demonstrated high temperature sensitivity and rapid response rates above the LCST of PNIPAM. NL hydrogel samples with a diameter of 28 mm were used for testing. As shown in Figure 3a, when heated at a rate of 0.6 °C/min, the NL hydrogels (especially those with higher PNIPAM/lignin ratios) exhibited a pronounced volumetric phase transition temperature (VPTT) near 33 °C. This transition is characterized by a sharp decrease in the volume ratio (V_T_/V_45_), where V_T_ and V_45_ represent the equilibrium-swollen volume of the hydrogel at temperature T and at 45 °C, respectively. In contrast, conventional PNIPAM hydrogels showed a gradual volume reduction as the temperature increased under identical heating conditions. Figure 3b illustrates the time-dependent water retention of the hydrogels after immersion in water at 45 °C. All the NL hydrogels exhibited rapid water loss: reaching approximately 90% within 10 s and achieving equilibrium in 30 s, regardless of the varying final shrinkage levels. In contrast, the water loss process of the PNIPAM hydrogel remained incomplete even after the long soaking time of 60 s. The volume shrinkage and response rate of the NL hydrogels surpass the values of most reported thermo-responsive hydrogels with high-volume change ratios and rapid responsive rates (Figure 3c). Furthermore, the dehydrated NL hydrogels regained most of their water at room temperature within 20 min (Figure S2). The slow response rate of the PNIPAM hydrogel is attributed to the formation of a dense surface layer during heating, which acts as a barrier preventing internal water from diffusing out [46]. Generally, the rapid response of and significant volume change in thermal-responsive hydrogels are achieved through the size effect (smaller scales lead to a faster response), and the presence of multi-water channels [27]. Based on this theory, NL hydrogels are expected to contain microstructures that facilitate their rapid response and significant volume shrinkage upon heating.

### 2.2. Structure of NL Hydrogels

**Figure 4:**
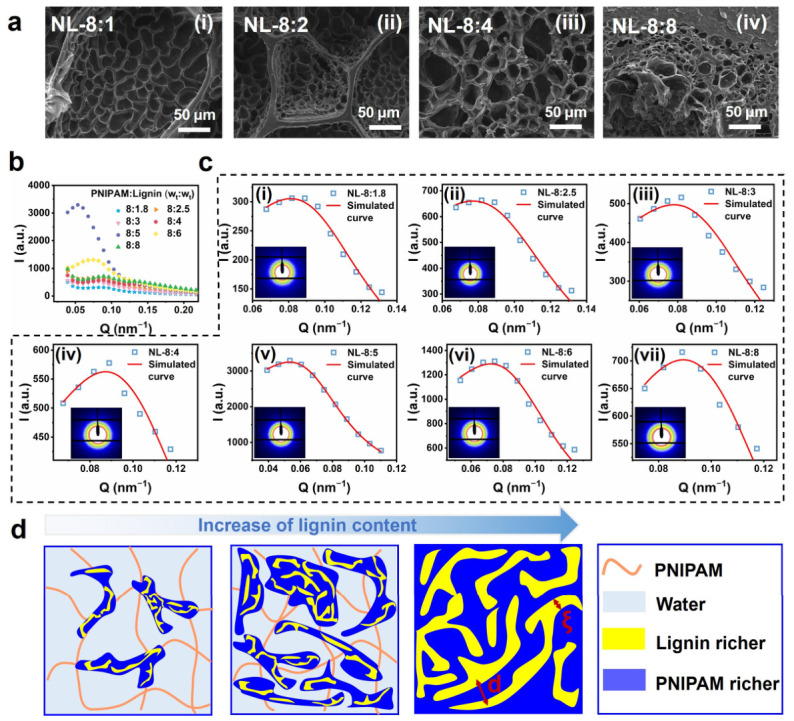
(**a**) The SEM images of the cross-sections of (i) NL_8:1, (ii) NL_8:2, (iii) NL_8:4, and (iv) NL_8:8 hydrogels. (**b**) one-dimensional SAXS profile. (**c**) The T-S model was used to fit the 1D curve diagram of (i) NL-8:1.8, (ii) NL-8:2.5, (iii) NL-8:3, (iv) NL-8:4, (v) NL-8:5 (vi) NL-8:6, and (vii) NL-8:8 hydrogels and (inset) the 2D SAXS pattern of (i) NL-8:1.8, (ii) NL-8:2.5, (iii) NL-8:3, (iv) NL-8:4, (v) NL-8:5 (vi) NL-8:6, and (vii) NL-8:8 hydrogels. (**d**) Schematic illustration of phase separation structures of NL hydrogels with the increase in lignin content.

To understand the rapid and high-volume changes in NL hydrogels, their microstructures at different observation scales were analyzed using SEM and SAXS, respectively. From Figure 4(ai,aii), the hierarchical structure of a mesh-like network with thin walls and a honeycomb-like skeleton with thick walls (scale > 10 μm) was observed in the SEM images of the freeze-dried NL_8:1 and 8:2 hydrogels. This demonstrated an inhomogeneous distribution of molecular chains with dense regions (skeletons) dispersed within a chain sparse sea (the mesh-like network). Considering the composition of the NL-8:1 and 8:2 hydrogel, where the lignin content was much lower than PNIPAM, and the similar mesh-like network observed in the pure PNIPAM hydrogel (Figure S3), we attributed the ‘mesh-like network’ to the swollen, PNIPAM-rich regions. The hydrophobic lignin, which cannot absorb much water, would be primarily located in chain-dense regions. The size of the ‘skeleton’ and the pores of the ‘mesh-like network’ decreased with the increase in the lignin content, indicating the higher crosslinking density and more chain-dense regions. When the PNIPAM/lignin ratio exceeded 8:4, the hierarchical structure disappeared in the SEM images, and was replaced by a more homogeneous porous structure with larger pore sizes and relatively thick walls. With the further increases in lignin content, the pores became smaller and the walls thicker, indicating a denser structure of the hydrogel.

SAXS measurements were performed to further explore the microstructure of NL hydrogels at a smaller observation scale. All of the 2D SAXS patterns of the NL hydrogels exhibited isotropic rings (Figure 4(ci–vii) (inset)), indicating the isotropic structure of these hydrogels. In the 1D SAXS profiles, the broad peaks with the peak positions in the range of 0.05–0.1 nm^−1^ were observed for all the NL hydrogels, indicating the existence of phase-separated microstructures (Figure 4b). According to the Bragg equation for long-period structures:

(1) $$l=2\pi /q_{\mathrm{peak}}$$

where $q_{\mathrm{peak}}$ is the peak position in the 1D SAXS profile, the long period of the scattering structure in the NL hydrogel was in the range of 70–120 nm. Since the PNIPAM phase was unlikely to form a phase-separated structure at room temperature, we attributed the scattering structure in the NL hydrogels to lignin-rich chain-dense regions. To further analyze this phase separation, the 1D SAXS profiles were fitted using the Teubner–Strey (T-S) model, which describes a bicontinuous phase structure [51,52,53]. As shown in Figure 4(ci–vii), the profiles were well-fitted by the T-S model. The periodicity (d) and correlation length $\left(\xi \right)$ from the fitting are shown in Table 1. At low lignin content, d and ξ remained almost unchanged with the lignin content, suggesting a consistent microstructure within the chain-dense region. When the PNIPAM/lignin ratio decreased to 8:5, d increased abruptly to 95 nm, and then gradually decreased with the decrease in the PNIPAM/lignin ratio. Based on the gel compositions, the volume contents of lignin ($\phi_{\mathrm{lg}}$) were estimated (Supporting Information). As the nanostructure of a bicontinuous phase structure is locally lamellar [51,52,53], the volume ratio of one phase ($\phi_{1}$) should be proportional to ξ/d, which reflects the connectivity and uniformity of the bicontinuous phase relative to its characteristic length scale. As shown in Table 1, ξ/d of all the gels was around 0.3, indecating the similar relative structural correlation and connectivity of the local bicontinuous structure. It was found that for the hydrogels with a PNIPAM/lignin ratio ≤ 8:5, $\phi_{\mathrm{lg}}/\left(\xi /d\right)$s values were all approximately 0.5 (Table 1). Considering the hydrophobicity of lignin, we assumed the lignin was mainly contained in the denser phase of the bicontinuous phase structure, and the lignin content of the denser phase was the same for all the NL hydrogels. Based on this assumption, $\phi_{\mathrm{lg}}/\left(\xi /d\right)$ should be constant if the hydrogel consists solely of a bicontinuous structure. The smaller $\phi_{\mathrm{lg}}/\left(\xi /d\right)$ reflects the mismatch between local structural features and the overall lignin distribution of the hydrogel, demonstrating that the bicontinuous domains are discretely dispersed in the network instead of forming a fully integrated continuous phase. Thus, the calculated results of Table 1 suggest that the NL hydrogels with a PNIPAM/lignin ratio ≤ 8:5 consisted mainly of chain-dense regions with a bicontinuous phase structure, while those with ratios > 8:5 contained both a chain-dense region and a PNIPAM-rich chain-sparse region, which is consistent with the SEM observations. At 45 °C, the scattering peak shifted to a higher q, corresponding to the smaller long-period distance caused by the dehydration of PNIPAM (Figure S4).

Thus, the structure of the NL hydrogels becomes clear. At low lignin content, the NL hydrogels possess a hierarchical phase-separated structure: lignin and a portion of PNIPAM form the chain-dense domains with a bicontinuous phase-separated structure, which are dispersed within the highly swollen PNIPAM phase and act as physical crosslinks. The chain-dense regions expand with the increase in the lignin content, eventually forming a fully bicontinuous structure at a ratio of 8:5 (Figure 4d). The bicontinuous structure of the chain-dense regions combined with the large mesh size in the chain-sparse region provides multi-scale water channels throughout the hydrogel. As the NL hydrogel is crosslinked by the chain-dense region, the PNIPAM in the chain-sparse region (lacking chemical crosslinks) retains high chain mobility. The multi-scale water channels and the high mobility of PNIPAM in the chain-sparse region synergistically enable the rapid response and significant volume shrinkage of the hydrogel. This structural evolution also explains the observed reduction in the volume change ratio: as the lignin content increases, the expanding chain-dense regions, which possess limited deformation capacity, occupy a larger volume fraction of the gel, thereby restricting its overall shrinkage capability.

### 2.3. Mechanical Properties and Energy Dissipation Mechanism of Lignin Hydrogels

**Figure 5:**
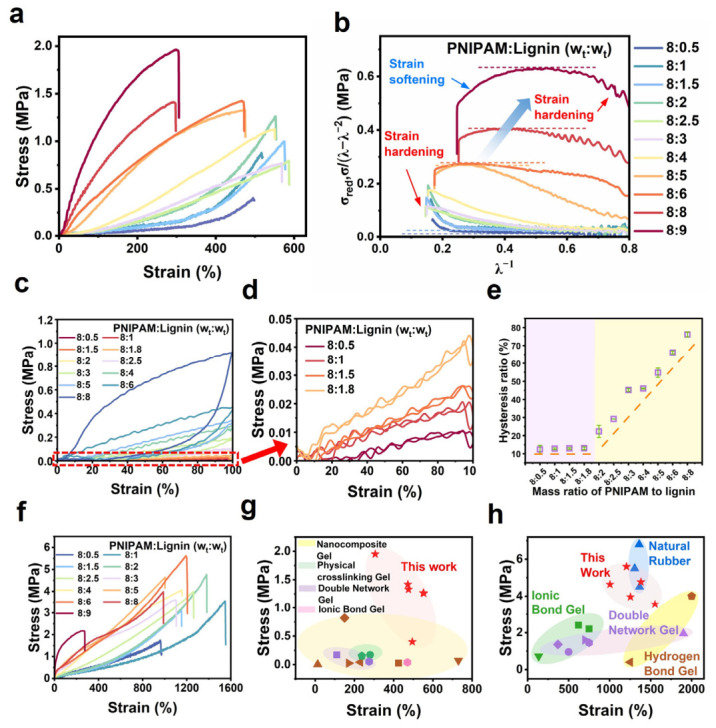
(**a**) Tensile stress–strain curves and (**b**) the related Mooney–Rivlin curves of NL hydrogels at room temperature. (**c**) Cyclic tensile test of the NL hydrogels under the same strain at room temperature. (**d**) Localized enlarged figure of (**c**). (**e**) Hysteresis ratio of NL hydrogels at room temperature. (**f**) Tensile stress–strain curves of NL hydrogels at 45 °C. (**g**) Comparison of tensile stress and strain of NL hydrogels with other thermo-responsive hydrogels at temperature below LCST [33,34,37,38,57,58,59,60,61,62]. (**h**) Comparison in the tensile stress and strain of the NL hydrogels above LCST with other tough hydrogels and natural rubber [15,36,54,63,64,65,66,67,68,69,70].

The structural transition of the NL hydrogels with the increase in lignin content also influenced their mechanical properties. As shown in Figure 5a, the tensile stress– strain curves of the NL hydrogels with a high lignin content (PNIPAM/lignin ratio ≤ 8:5) all showed an unobvious yield in intermediate strain conditions. In contrast, the NL gels with a lower lignin content (PNIPAM/lignin ratio > 8:5) did not exhibit yielding during the tensile test. The tensile behavior of the NL hydrogels was further analyzed using the phenomenological Mooney–Rivlin equation [54,55,56], which is expressed as follows:

(2) $$\sigma_{\mathrm{red}}=\frac{\sigma }{\lambda -\lambda^{-2}}=2C_{1}+2C_{2}\frac{1}{\lambda }$$

where $\sigma_{\mathrm{red}}$ is the reduced stress, λ is the stretch ratio, and $C_{1}$ and $C_{2}$ are the material constants. $2C_{1}$ is equal to the shear modulus (≈E/3). The negative $C_{2}$ value is related to strain hardening, and positive $C_{2}$ indicates strain softening beyond the Gaussian elasticity region. $C_{2}$ = 0 means the material has a purely elastic stretching region.

As shown in Figure 5b, $\sigma_{\mathrm{red}}$ of the NL hydrogels with a PNIPAM/lignin ratio > 8:5 remained almost constant across a wide range of stretch ratios, demonstrating the pure elasticity of these gels. In these hydrogels, the chain-dense regions act as crosslink points dispersed in the PNIPAM-rich chain-sparse ‘sea’. Consequently, the entropic stretching of the PNIPAM random coils in the chain-sparse regions dominated the mechanical properties of the hydrogel from the small to moderate stretch ratios. When the entire hydrogel formed a bicontinuous phase-separated structure (PNIPAM/lignin ratio ≤ 8:5), the highly restricted mobility of the polymer chains caused strain hardening ($C_{2}$ < 0), to much lower stretch ratios. This was subsequently followed by fracture-induced strain softening ($C_{2}$ > 0). Due to the relatively high rigidity of the denser regions in the bicontinuous phase, the initial $\sigma_{\mathrm{red}}$ increased with the further increase in lignin content in the NL hydrogels, and the yielding point—marking the onset of strain softening—shifted to a lower stretch ratio.

Differences in mechanical properties were also observed in the cyclic tensile test of the NL hydrogels. As shown in Figure 5c,d, at a low lignin content (PNIPAM/lignin ratio > 8:2), the NL hydrogels were nearly purely elastic, with negligible energy dissipation and minimal hysteresis ratios were evident. When the PNIPAM/lignin ratio decreased to 8:3, energy dissipation emerged due to the deformation of the chain-dense regions. The hysteresis ratio increased abruptly when the PNIPAM/lignin ≤ 8:5, which continually increased with the further decrease in the PNIPAM/lignin ratio.

As chain-dense regions can significantly dissipate energy and act as multi-functional crosslink points, the NL hydrogels all exhibited excellent mechanical properties, with high tensile strengths of around MPa (Figure 5a). In contrast, the tensile strength of the PNIPAM hydrogel was only 0.02 MPa at 25 °C (Figure S5). After heating, the highly increased chain density further improved the mechanical properties of the NL hydrogels. As shown in Figure 5f, the tensile stress of all the NL hydrogels was above 1 MPa, and the NL-8:6 hydrogel showed super-high mechanical properties with tensile stress and strain values up to ~6 MPa and 12, respectively. The comparison with literature-reported thermo-responsive hydrogels demonstrates that the NL hydrogels exhibit superior mechanical properties below the LCST of PNIPAM (Figure 5g). After heating to a temperature above the LCST of PNIPAM, the mechanical properties of NL hydrogels are superior to many reported tough hydrogels and comparable to those of natural rubber (Figure 5h). The composition and performance of NL hydrogels were compared with previously reported lignin-based stimuli-responsive hydrogels (Table S1). This comparison highlights the superior mechanical strength and rapid actuation of our system.

The rapid response, significant volume change, and high mechanical strength create a high actuation force within a short time, which is superior to many reported responsive hydrogels (Figure S6), thus providing its great application potential as a hydrogel actuator.

### 2.4. Controlled Deformation of Printed Lignin Hydrogel Actuators

**Figure 6:**
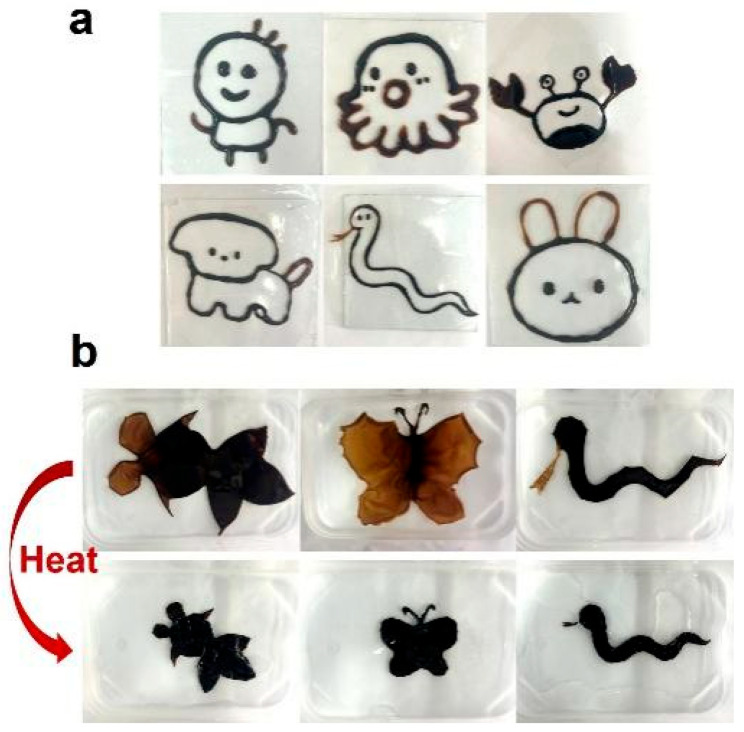
(**a**) Various NL hydrogel patterns printed by the direct ink writing method. (**b**) The photos of the printed goldfish, butterfly, and snake patterns with the body of the NL-8:6 hydrogel and eyes, wings, and tongue of the NL-8:1.5 hydrogel before and after heating.

NL hydrogels exhibited printability due to their straightforward precursor drying and swelling process. Using DIW technology, various complex geometries, such as an octopus and a dog, were successfully printed (Figure 6a, Movie S1). By integrating different NL hydrogel grades (NL-8:1.5 for appendages and NL-8:6 for bodies), as shown in Figure 6b, models with site-specific responsiveness were created. For instance, ‘butterfly wings’ and ‘snake tongues’ were initially enlarged at 25 °C, but underwent noticeable shrinking upon heating.

**Figure 7:**
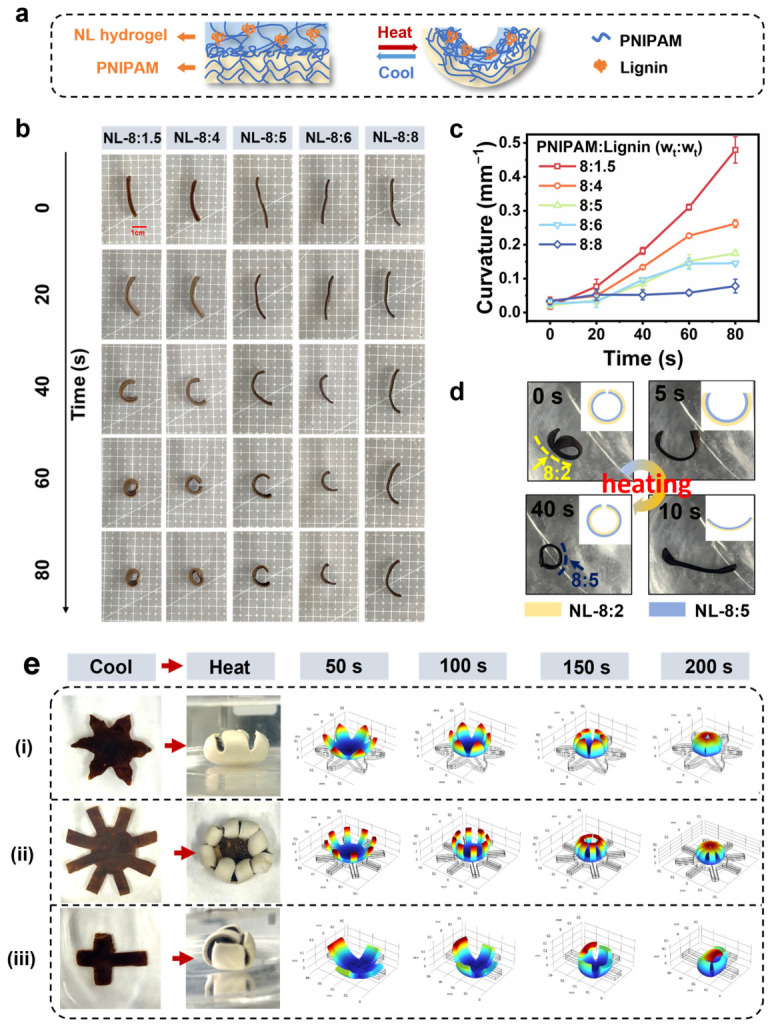
The influence of lignin content on the dynamic thermo-responsive bending and shrinking behaviors of bilayer hydrogel strips at 45 °C: (**a**) schematic illustration of bilayer hydrogels’ preparation and their reversible structural transformation; (**b**) the optical images demonstrate the dynamic process; (**c**) dynamic changes in the curvature of bilayer hydrogel strips with different lignin contents. (**d**) Photos and schematic illustrations (insets) of the reverse bending process of the bilayer hydrogel strips prepared from NL hydrogels with PNIPAM to lignin ratios of 8:2 and 8:5 in 45 °C water. (**e**) Photographs of the thermo-responsive actuators with the shape of (i) a 6-arm star, (ii) gearwheel, and (iii) cross before and after being heated, and their stress simulation analysis after heating.

By printing NL hydrogels with different lignin contents on a hydrogel with a different volume change ratio after heating, a thermo-responsive hydrogel actuator could be easily fabricated. As demonstrated in Figure 7a,b, NL/PNIPAM bilayer actuators obtained by printing NL hydrogels on PNIPAM hydrogel strips exhibit directional bending toward the NL hydrogel side in water at 45 °C, due to the higher volume shrinkage ratios of the NL hydrogels. Owing to the tunable volume shrinkage ratio of the NL hydrogel layer, the NL/PNIPAM hydrogel actuators with varying NL hydrogel layers exhibit distinct bending properties (Figure 7b). At 60 s, the NL-8:1.5/PNIPAM actuator curled significantly, forming a closed ring. The curvature of the NL/PNIPAM actuator gradually decreased with the increase in lignin content of the NL layer, and the NL-8:8/PNIPAM actuator only slightly bent after being immersed in 45 °C water for 80 s (Figure 7b). Figure 7c presents the dynamic thermo-responsive curvature profiles of these NL/PNIPAM hydrogel actuators in water at 45 °C. The NL-8:1.5 PNIPAM actuator demonstrated the highest curvature variation, reaching a value as high as 0.48 mm^−1^. As the lignin content increases, the curvature values decrease progressively. All the NL/PNIPAM bilayer actuators completed their bending within 80 s. Furthermore, the hydrogel bilayer actuator composing NL hydrogel layers with different lignin content exhibited significant and rapid reverse bending within 40 s upon immersion in 45 °C water (Figure 7d, Movie S2). While many biopolymer-based 4D-printed materials, especially the ones triggered by hydration/dehydration and/or temperature, suffered from prolonged response times [45], the NL hydrogel system exhibited distinct competitive advantages for practical applications.

The geometric design can influence the stress distribution within a hydrogel actuator. Thus, by designing the shapes of the NL/PNIPAM bilayer hydrogel, hydrogel actuators with different actions can be developed. As shown in Figure 7(ei), the 6-arm star-shaped NL-8:1.5/PNIPAM bilayer hydrogel exhibited stress concentration at the arm tip upon heating, triggering inward bending to form a functional claw. Increasing the tip width to a gearwheel shape (Figure 7(eii)) expanded the stress-concentrated area, thereby enhancing the bending force and leading to complete arm curvature. Similarly, a cross-shaped configuration (Figure 7(eiii)), directed the stress concentration to the longer arm, facilitating the transformation into a cube. Furthermore, the DIW allows for more complex shape transitions by varying the lignin content within NL hydrogel layers. As shown in Figure S7iii,iv, disk-shaped PNIPAM hydrogels with an NL-8:1.5 center and NL-8:6 outer ring and vice versa transformed from a saddle shape to a Chinese cabbage shape and a dumpling shape upon heating, respectively.

The tunable curvature characteristics, combined with high curvature and printable capability, make NL hydrogels highly designable, highlighting their advantages for actuators and manipulators in soft robots.

### 2.5. Application of NL Hydrogels

**Figure 8:**
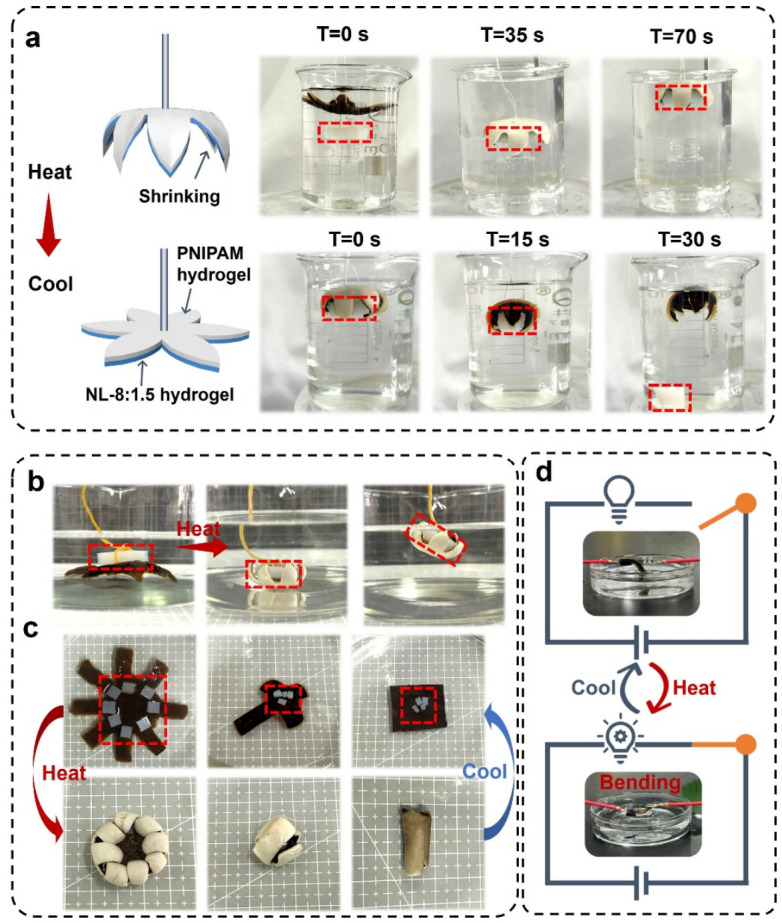
(**a**) Photos and schematics of the bilayer hydrogel gripper capturing a silicone block in water at 45 °C and releasing it at 25 °C. (**b**) Transportation of a bilayer hydrogel with a six-arm star shape by a silicone block, like fishing. (**c**) The reversible wrapping, encapsulation, and release of silicone blocks in thermo-responsive bilayer hydrogels with different shapes. (**d**) Soft switching of hydrogel actuators controlled by temperature change.

The application of the NL/PNIPAM bilayer hydrogel, with a 6-arm star shape, as an underwater manipulator was first investigated. As shown in Figure 8a, the manipulator can close and grasp a silicone block within 70 s upon heating to 45 °C (Movie S3), subsequently releasing it within 30 s as the water cools to 25 °C. Such an NL/PNIPAM bilayer hydrogel can also be ‘fished up’ by silicon block ‘bait’ when the water temperature increases (Figure 8b). Beyond the star shape, bilayer hydrogels in gearwheel, cross, and rectangular geometries demonstrated versatile encapsulation and the release of silicone targets (Figure 8c), highlighting their potential for drug delivery and cargo transportation. Due to the intrinsic ionic conductivity of hydrogels, the bilayer hydrogel was also used as a thermal soft switch. As shown in Figure 8d, at a temperature below the LCST, the hydrogel actuator remained detached; once heated above the LCST, it bent to contact the electrode, completing the circuit, and illuminating a LED (Figure S8).

**Figure 9:**
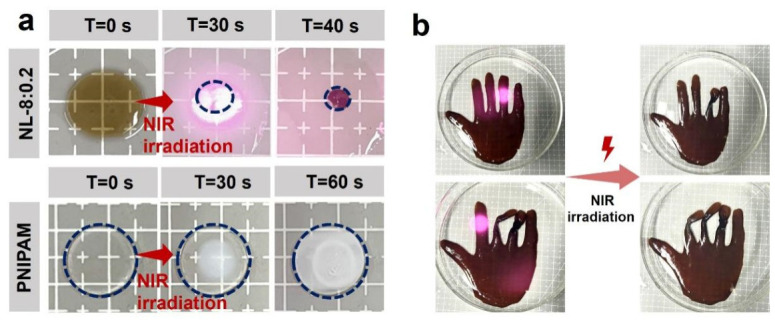
(**a**) Optical images of the dynamic response process of NL and PNIPAM hydrogel discs to NIR irradiation. (**b**) Images of the fingers of hand-shaped hydrogel bending in response to the location of an NIR laser spot.

Generally, traditional temperature-driven hydrogel actuation suffers from the difficulty in achieving precise and localized temperature control, which limits accurate complex shape-morphing performance. To address such spatiotemporal resolution bottlenecks, photochemically tuning the LCST has emerged as an effective alternative strategy for PNIPAM-based systems [71,72]. The intrinsic photothermal effect of lignin enabled the NL hydrogel to respond rapidly to the near infrared (NIR) quite fast. Upon NIR exposure at room temperature, the NL-8:0.2 hydrogel disc exhibited significant volumetric shrinkage within 40 s (Figure 9a, Movie S4). In contrast, the pure PNIPAM hydrogel only showed localized, uneven whitening after 60 s, without observable volume change even after 4 min of continuous NIR irradiation (Figure 9a, Movie S4). Leveraging this sensitivity, a hand-shaped NL hydrogel was fabricated to demonstrate irradiation-controlled actuation. By sequentially directing the laser spot onto the ‘fingers,’ localized shrinkage was induced, triggering bending movements (Figure 9b).

## 3. Conclusions

In summary, novel lignin/PNIPAM thermo-responsive hydrogels (NL hydrogels) featuring a hierarchical phase-separated structure have been successfully developed via a facile drying–swelling process. The internal architecture depended on the composition: at a low lignin content, the system self-assembled into lignin-rich, dense domains with a bicontinuous phase-separated structure dispersed within a PNIPAM-rich, chain-sparse matrix. The multi-scale water channels within the hierarchical phase-separated structure and high chain mobility within these sparse regions resulted in a large volume change ratio and ultrafast response. As the lignin content increased, these chain-dense domains expanded, leading to a decreased swelling ratio while providing a tunable mechanism for performance modulation. Furthermore, these dense domains act as efficient energy-dissipation units, endowing the NL systems with superior mechanical strength that outperforms most thermo-responsive hydrogels. Direct ink writing (DIW) was employed to fabricate complex geometries, leveraging the material’s excellent processability. Multi-material printing of NL/PNIPAM bilayers yielded actuators with tunable curvatures and versatile locomotion (e.g., grabbing and wrapping). Additionally, the photothermal effect of lignin endows the NL hydrogel with a faster response to NIR light. These NL hydrogels not only resolve the trade-off between mechanical strength and responsiveness, but also overcome the slow actuation of 4D-printable biopolymers. This combination highlights their significant potential for soft robotics and sustainable smart structures.

## 4. Materials and Methods

### 4.1. Materials

Guangzhou Yinnovator Biotech Co., Ltd., Guangzhou, China, supplied acetic acid lignin (S/G = 1.05, Mw of 5.9 kDa, PDI of 1.6, determined by the polystyrene calibration curve) from bamboo. The lignin was vacuum-dried at 60 °C for 24 h prior to use. N-Isopropylacrylamide (NIPAM) was obtained from Shanghai Aladdin Bio-Chem Technology Co., Ltd., Shanghai, China. α-Ketoglutaric acid (AR > 98%) was received from Shanghai Macklin Biochemical Co., Ltd., Shanghai, China. N, N-Dimethylformamide (DMF) was sourced from Tianjin Fuyu Fine Chemical Co., Ltd., Tianjin, China. N, N-methylenebis (acrylamide) (MBAA) was provided by Tianjin Kemiou Chemical Co., Ltd., Tianjin, China. All reagents were used as received.

### 4.2. Synthesis of NL Hydrogels

A 10 mL aliquot of NIPAM aqueous solution (1 wt%) containing alpha-ketoglutaric acid (0.2 mol% of NIPAM) was polymerized under 365 nm UV light (≈70 mW cm^−2^) in a sealed syringe, yielding the PNIPAM solution. The solution was freeze-dried using an FD-1A-50 freeze-drier (Shanghai Oumeng Industrial Co., Ltd., Shanghai, China) for 48 h to complete dryness, obtaining solid PNIPAM. The solid PNIPAM and various amounts of lignin were separately dissolved in DMF to prepare a 7.8 wt% PNIPAM/DMF solution and 0.3–21.2 wt% (corresponding to a PNIPAM/lignin mass ratio range of 8:0.1–8:9) lignin/DMF solution. These two solutions were then mixed to form a homogeneous PNIPAM/lignin precursor solution, which was also directly used as the 4D printing ink. The precursor solution was poured into silicone molds and dried at room temperature (25 °C) to a constant weight. The resulting dried sheet was immersed in a large amount of water for over 24 h to reach the equilibrium swelling state according to the swelling equilibrium curve of the dry sheet in water (Figure S9). The PNIPAM/lignin hydrogel (NL hydrogel) was then obtained. The NL hydrogels were coded as NL-a:b, where a:b represents the mass ratio of PNIPAM to lignin, ranging from 8:0.1 to 8:9.

### 4.3. Synthesis of Covalently Crosslinked PNIPAM Hydrogels

A total of 10 mL of NIPAM precursor aqueous solution with NIPAM (10 wt%), α-ketoglutaric acid (0.2 mol% of NIPAM), and MBAA (1 mol% of NIPAM) was injected into a reaction cell (80 × 80 × 1.5 mm) with a pair of glass substrates separated by a 0.9 mm-thick silicone spacer. The sample was exposed to 365 nm UV light (≈70 mW cm^−2^) for 10 h to produce the PNIPAM hydrogel.

### 4.4. Preparation of Bilayer Hydrogel Actuator

The PNIPAM/lignin precursor solution was printed onto a covalently crosslinked PNIPAM hydrogel by direct handwriting with a syringe. Upon complete drying, the sample was immersed in water, yielding PNIPAM/NL hydrogels with a bilayer structure. The printing amount of the precursor solution on the PNIPAM hydrogel determined the thickness of the NL hydrogel layer.

### 4.5. Preparation of Bilayer NL Hydrogel

The dried NL-8:2 and NL-8:5 sheets were stacked, and the PNIPAM/lignin DMF solution was applied dropwise at the interface. Pressure was then applied to ensure tight bonding. After drying, the sample was immersed in water for swelling, yielding a bilayer NL hydrogel.

### 4.6. Calculation of the Water Content Measurement

The swollen NL hydrogel samples were cut into discs with a diameter of 28 mm. After removing surface water, their mass was recorded as $m_{a}$. The samples were then heated in 45 °C water until complete shrinkage, and the mass was recorded as $m_{b}$. Both the swollen and shrinking samples were then freeze-dried (FD-1A-50, Shanghai Oumeng Industrial Co., Ltd.) for 48 h to obtain the dried hydrogel samples; their mass was recorded as $m_{d}$. The water content of swollen samples was calculated as follows:

(3) $$C_{\mathrm{wt}}=\frac{\left(m_{a}-m_{d}\right)}{m_{a}}\times 100\%$$

The water content of shrinking samples was calculated as follows:

(4) $$C_{\mathrm{wt}}=\frac{\left(m_{b}-m_{d}\right)}{m_{b}}\times 100\%$$

### 4.7. Calculation of the Volume Shrinkage

The swollen NL hydrogel was cut into 13 mm discs and then placed in 45 °C water until complete shrinkage. The diameters and thicknesses of both the swollen and shrinking states were measured. The volume shrinkage was calculated as follows:

(5) $$\mathrm{Volume}\;\mathrm{shrinkage}=\frac{V_{a}-V_{b}}{V_{a}}\times 100\%$$

where $V_{a}$ is the volume of the hydrogel in the swollen state, and $V_{b}$ is the volume after heating and shrinking.

### 4.8. Thermo-Responsive Kinetic Studies of NL Hydrogels

(1) Volume Ratio Measurements:

The fully swollen NL hydrogel was cut into discs with a diameter of 28 mm, and the surface water was removed with filter paper. The hydrogel samples were then placed in beakers, and the temperature of the water bath was controlled using a heated magnetic stirrer (Model: SCI340-Pro). During the experiment, the temperature was increased from 25 °C to 45 °C at a heating rate of 0.6 °C/min. The volume of the samples was determined at every 5 °C interval. Volume ratio was calculated using the following formula:

(6) $$\mathrm{Volume}\;\mathrm{ratio}=\frac{V_{T}}{V_{d}}$$

where $V_{T}$ is the equilibrium volume of the hydrogel at temperature T, and $V_{d}$ is the volume of the hydrogel in the dry state.

(2) Deswelling Kinetic Measurements:

The swelling-equilibrated NL hydrogels were cut into 28 mm discs and immersed in 45 °C water to reach shrinkage equilibrium. During this process, the hydrogel samples were taken out successively every 5 s to measure the weight. The water on the surface of Each sample was wiped before weighed. Water retention was calculated by:

(7) $$\mathrm{Water}\;\mathrm{retention}=\frac{W_{t}-W_{d}}{W_{0}-W_{d}}\times 100\%$$

where $W_{t}$ is the weight of a wet hydrogel at regular time intervals, $W_{0}$ is the weight of equilibrium hydrogel at room temperature, and $W_{d}$ is the dry weight of the hydrogel.

(3) Reswelling Kinetic Measurements:

The hydrogel discs in the state of shrinkage equilibrium at 45 °C were placed in water at 25 °C to swell until swelling equilibrium was reached. During this process, the hydrogel samples were taken out successively every 5 min to measure the weight. The water on the surface of each sample was wiped before being weighed. The water uptake was calculated using the following formula:

(8) $$\mathrm{Water}\;\mathrm{uptake}=\frac{W_{t}-W_{d}}{W_{0}-W_{d}}\times 100\%$$

where $W_{t}$ is the weight of a wet hydrogel at regular time intervals, $W_{0}$ is the weight of the equilibrium hydrogel at room temperature, and $W_{d}$ is the dry weight of the hydrogel.

### 4.9. Scanning Electron Microscopy (SEM) Test

The SEM test of hydrogels was performed using a Vega 3 SBH scanning electron microscope at an acceleration voltage of 12.0 kV. Samples were prepared by cryogenic fracturing in liquid nitrogen followed by a freeze-drying process (FD-1A-50, Shanghai Oumeng Industrial Co., Ltd.). The fractured surface was covered with a thin layer of gold using the sputtering method before observation.

### 4.10. Small-Angle X-Ray Scattering (SAXS) Test

SAXS measurements were conducted on lignin hydrogels using BL19U2 beamline at the Shanghai Synchrotron Radiation Facility (SSRF, China), with a camera length of 5713 mm and X-ray wavelength of 1.03 Å. The 2D SAXS patterns were recorded using a PILATUS × 2M detector with a 1475 × 1679 pixel resolution and a 172 μm pixel size.

### 4.11. Calculation of d and ξ Based on the Teubner–Strey (T-S) Model

The expression of the T-S model is as follows:

(9) $$I\left(q\right)=\frac{8\pi {\left(\Delta \rho \right)}^{2}}{\xi }\frac{1}{a_{2}+c_{1}q^{2}+c_{2}q^{4}}$$

where $a_{2}$, $c_{1}$, and $c_{2}$ are coefficients based on the Ginzburg–Landau theory. The two parameters d and ξ, which stand for periodicity and persistence length or correlation length, respectively, can be expressed as follows:

(10) $$d=2\pi {\left[\frac{1}{2}{\left(\frac{a_{2}}{c_{2}}\right)}^{1/2}-\frac{1}{4}\frac{c_{1}}{c_{2}}\right]}^{-1/2}$$

(11) $$\xi ={\left[\frac{1}{2}{\left(\frac{a_{2}}{c_{2}}\right)}^{1/2}+\frac{1}{4}\frac{c_{1}}{c_{2}}\right]}^{-1/2}$$

### 4.12. Calculation of the Volume Contents of Lignin ($\phi_{lg}$)

(12) $$\phi_{lg}=\frac{V_{lignin}}{V_{lignin}+V_{PNIPAM+water}}$$

where $\rho_{\mathrm{lignin}}$ is taken as 1.3 g/cm^3^, $V_{\mathrm{lignin}}$ is calculated using the following formula:

(13) $$V_{\mathrm{lignin}}=\frac{m_{\mathrm{lignin}}}{\rho_{\mathrm{lignin}}}$$

$\rho_{\mathrm{PNIPAM}+\mathrm{water}}$ were calculated as 1.0 g/cm^3^; $V_{\mathrm{PNIPAM}+\mathrm{water}}$ was calculated using the following formula:

(14) $$V_{\mathrm{PNIPAM}+\mathrm{water}}=\frac{m_{\mathrm{PNIPAM}}+m_{\mathrm{water}}}{\rho_{\mathrm{PNIPAM}+\mathrm{water}}}$$

### 4.13. Tensile Test

Dumbbell-shaped specimens of JIS-K6215-7 standard size (12 mm gauge length × 2 mm width × ≈ 0.4 mm thickness) were subjected to uniaxial tensile tests in water at 25 °C and 45 °C, respectively, using an HZ-1007C electronic universal testing machine (Dongwanlixian Co., Ltd., Dongwan, China). The initial distance between fixtures L_0_ was 12 mm, the tensile deformation rate was 0.14 s^−1^, and the load sensor was 50 N. The work of extension at fracture W is defined as the area under the stress–strain curve. The hysteresis ratio is defined as the ratio of the area between the loading and unloading curves to the area under the loading curve (Figure S10). Loading–unloading tests were also conducted at the same stretch velocity and sample size. Each sample was first stretched to a strain of ε = 1, then immediately returned to the initial displacement at the same stretch velocity.

### 4.14. Direct Ink Writing (DIW) Method

The NL hydrogel precursor solution was loaded into a 10 mL syringe equipped with a stainless-steel needle (inner diameter: 0.84 mm). The syringe was held by hand, and pressure was applied to the plunger to manually extrude the precursor solution. Following a predesigned pattern, the extruded hydrogel filament was directly deposited onto a glass plate. After extrusion, the printed hydrogel pattern was completely dried at room temperature and then immersed in deionized water to swell for 12 h, yielding hydrogels with different shapes. All manual printing operations were performed at room temperature (25 °C).

### 4.15. Thermo-Responsive Bending Behavior of NL/PNIPAM Bilayer Hydrogels

NL/PNIPAM bilayer hydrogels with different lignin contents were cut into strips measuring 5 mm in width and 30 mm in length, then immersed in 45 °C water. Before the dynamic thermo-responsive bending test, the bilayer hydrogel strips were maintained in 25 °C water for 24 h to reach complete swelling equilibrium. The entire dynamic bending process was recorded using a digital camera. The curvature of the bilayer hydrogels was calculated as follows:

(15) $$\mathrm{Curvature}=\frac{1}{R}$$

R is the bending radius (mm), obtained by fitting the geometric profile of the curved arc.

### 4.16. Stress Simulation Analysis

COMSOL 6.2 was used to perform numerical simulations on the mechanical response of the double layer. The fields of solid mechanics and porous elasticity were incorporated into COMSOL to simulate the deformation response of the structure. The Neo-Hookean model was chosen to describe the mechanical behavior of soft materials. To reduce the computational load, symmetry constraints were applied to the model.

### 4.17. Grasping, Transportation, and Encapsulation Behaviors of NL/PNIPAM Bilayer Hydrogels

Firstly, the equilibrium-swollen bilayer hydrogel with 6-arm star shape was hung by a string. The bilayer hydrogel was then placed on a silicone block submerged in water at 45 °C. Upon the thermal-induced shrinkage of its ‘arms’, the silicone block was captured by pulling the hydrogel. The bilayer hydrogel was then transferred back to 25 °C water to release the silicone block. The entire process from capture to release was captured by a digital camera. Secondly, a silicone block connected to a string was used as bait to fish the 6-arm star-shaped NL/PNIPAM bilayer hydrogel. Upon thermal shrinkage, the silicone block became tightly bit by the bilayer hydrogel. The entire transport process was recorded using a digital camera. Thirdly, the NL/PNIPAM bilayer hydrogel with the shapes of a gearwheel, cross, and rectangle were placed in 45 °C water to wrap and encapsulate small silicone blocks. As the bilayer hydrogel underwent thermal shrinkage, the small silicone blocks were wrapped up and encapsulated within the hydrogel. The process was recorded with a digital camera.

### 4.18. Near-Infrared Photothermal Response of the NL Hydrogel

The swollen NL hydrogel was cut into 13 mm diameter discs and irradiated with an 808 nm near-infrared laser (Model: BOT808-80B5) at a power setting of ~0.4 W. The power density was approximately 0.5 W/cm^2^, and the distance between the laser output port and the sample surface was 15 cm. All experiments were conducted at room temperature (25 °C). the NL hydrogel was shaped into a hand-like shape, where the irradiation of the joint areas induced finger-like bending motions.

## Figures and Tables

**Table 1:** Periodicity d; correlation length ξ, ξ/d of the bicontinuous phase structure; volume contents of lignin ($\phi_{\mathrm{lg}}$); and $\phi_{\mathrm{lg}}/(\xi /d)$ values of NL hydrogels with different PNIPAM/lignin ratios.

	NL-8:1.8	NL-2.5	NL-8:3	NL-8:4	NL-8:5	NL-8:6	NL-8:8
d	66.69	67.90	68.26	63.07	95.57	73.31	62.75
ξ	20.95	19.59	20.41	20.78	25.91	20.95	21.98
ξ/d	0.31	0.29	0.30	0.33	0.27	0.29	0.35
$\phi_{\mathrm{lg}}$	0.02	0.04	0.05	0.07	0.13	0.16	0.19
$\phi_{\mathrm{lg}}/(\xi /d)$	0.08	0.14	0.16	0.21	0.48	0.55	0.54

## Data Availability

The data that support the findings of this study are available from the corresponding authors upon reasonable request.

## Supplementary Materials

The following supporting information can be downloaded at: https://www.mdpi.com/article/10.3390/gels12050362/s1. Figure S1: Aqueous Solution of PNIPAM/lignin; Figure S2: Water uptake rate of the heating-induced shrunk NL hydrogels; Figure S3: The SEM image of the cross-section of the PNIPAM hydrogel; Figure S4: The 1D SAXS profile of the NL-8:4 gel at 25 °C and 45 °C; Figure S5: Tensile stress-strain curves of chemically crosslinked PNIPAM hydrogels: (a) at 25 °C and (b) at 45 °C; Figure S6: Comparison in the Actuation performance of NL hydrogel with other responsive hydrogel actuators [73,74,75,76,77,78,79,80]; Figure S7: Shape deformation of the NL hydrogel disk printed with different NL hydrogels: (a) the schematic illustration of the composition of the hydrogel disks (yellow for NL-8:1.5 gel, blue for NL-8:6 gel, and pink for PNIPAM gel); (b) the photos of printed hydrogels at the swollen state in 25 °C water; (c) the photos of the hydrogels after being heated to 45 °C; Figure S8: Soft switching of NL/PNIPAM bilayer hydrogel actuators controlled by temperature; Figure S9: Swelling equilibrium curve of NL hydrogels (w_d_ is the dry weight of the hydrogel, and w_s_ is calculated as the weight of the equilibrium-swollen hydrogel at regular time intervals minus w_d_); Figure S10: Schematic diagram illustrating the calculation of (a) work of extension at fracture (*W*) and (b) hysteresis area; Table S1: Comparison of Composition and Properties of NL Hydrogels and Lignin-Based Stimuli-Responsive Hydrogels [81,82,83,84,85,86].

## References

[1] Kim Y., Yuk H., Zhao R., Chester S.A., Zhao X. (2018). Printing ferromagnetic domains for untethered fast-transforming soft materials. Nature.

[2] Majidi C. (2019). Soft-Matter Engineering for Soft Robotics. Adv. Mater. Technol..

[3] Truby R.L. (2021). Designing Soft Robots as Robotic Materials. Acc. Mater. Res..

[4] Gu G., Zou J., Zhao R., Zhao X., Zhu X. (2018). Soft wall-climbing robots. Sci. Robot..

[5] Li W., Guan Q., Li M., Saiz E., Hou X. (2023). Nature-inspired strategies for the synthesis of hydrogel actuators and their applications. Prog. Polym. Sci..

[6] Ni C., Chen D., Wen X., Jin B., He Y., Xie T., Zhao Q. (2023). High speed underwater hydrogel robots with programmable motions powered by light. Nat. Commun..

[7] Koetting M.C., Peters J.T., Steichen S.D., Peppas N.A. (2015). Stimulus-responsive hydrogels: Theory, modern advances, and applications. Mater. Sci. Eng. R Rep..

[8] Sershen S.R., Mensing G.A., Ng M., Halas N.J., Beebe D.J., West J.L. (2005). Independent Optical Control of Microfluidic Valves Formed from Optomechanically Responsive Nanocomposite Hydrogels. Adv. Mater..

[9] Qin H., Zhang T., Li N., Cong H.-P., Yu S.-H. (2019). Anisotropic and self-healing hydrogels with multi-responsive actuating capability. Nat. Commun..

[10] Hua L., Xie M., Jian Y., Wu B., Chen C., Zhao C. (2019). Multiple-Responsive and Amphibious Hydrogel Actuator Based on Asymmetric UCST-Type Volume Phase Transition. ACS Appl. Mater. Interfaces.

[11] Chan A., Neufeld R. (2009). Modeling the controllable pH-responsive swelling and pore size of networked alginate based biomaterials. Biomaterials.

[12] Yang C., Wang W., Yao C., Xie R., Ju X.-J., Liu Z., Chu L.-Y. (2015). Hydrogel Walkers with Electro-Driven Motility for Cargo Transport. Sci. Rep..

[13] Haider H., Yang C., Zheng W., Jianhai Y., Wang M., Zrinyi M., Yang S., Osada Y., Suo Z., Zhang Q. (2015). Exceptionally Tough and Notch-Insensitive Magnetic Hydrogels. Soft Matter.

[14] Yuk H., Lin S., Ma C., Takaffoli M., Fang N.X., Zhao X. (2017). Hydraulic hydrogel actuators and robots optically and sonically camouflaged in water. Nat. Commun..

[15] Li Y., Liu L., Xu H., Cheng Z., Yan J., Xie X.-M. (2022). Biomimetic Gradient Hydrogel Actuators with Ultrafast Thermo-Responsiveness and High Strength. ACS Appl. Mater. Interfaces.

[16] Gao G., Wang L., Cong Y., Wang Z., Zhou Y., Wang R., Chen J., Fu J. (2018). Synergistic pH and Temperature-Driven Actuation of Poly(NIPAM-*co*-DMAPMA)/Clay Nanocomposite Hydrogel Bilayers. ACS Omega.

[17] Li S., Yang H., Zhu N., Chen G., Miao Y., Zheng J., Cong Y., Chen Y., Gao J., Jian X. (2023). Biotissue-Inspired Anisotropic Carbon Fiber Composite Hydrogels for Logic Gates, Integrated Soft Actuators, and Sensors with Ultra-High Sensitivity. Adv. Funct. Mater..

[18] Yao C., Liu Z., Yang C., Wang W., Ju X.-J., Xie R., Chu L.-Y. (2015). Poly(N-isopropylacrylamide)-Clay Nanocomposite Hydrogels with Responsive Bending Property as Temperature-Controlled Manipulators. Adv. Funct. Mater..

[19] Lu Z., Cui J., Liu F., Liang C., Feng S., Sun Y., Gao W., Guo Y., Zhang B., Huang W. (2024). A 4D Printed Adhesive, Thermo-Contractile, and Degradable Hydrogel for Diabetic Wound Healing. Adv. Healthc. Mater..

[20] Wang P., Lv Y., Duan J., Sun G., Meng C., Li Y., Guo S., Zhang T. (2025). A thermally responsive phase-change hydrogel for skin-mountable multifunctional sensors. Nano Energy.

[21] Yi Z., Song Z., Wang J., He M. (2024). Thermal and solvent responsive hydrogels for active–passive dual-control smart windows. Cellulose.

[22] Timusk M., Locs J., Kangur T., Kasikov A., Kurnitski J., Šutka A. (2023). Surface-Active Thermally Responsive Hydrogels by Emulsion Sedimentation for Smart Window Applications. ACS Appl. Polym. Mater..

[23] Zhao L.-L., Shi X.-L., Huang C.-H., Zou M.-L., Liang M.-Y., Liang J., Kang S.-M., Miao L., Chen Z.-G. (2025). Temperature-sensitive ionic hydrogels for dual electric and thermal responsive smart window. Chem. Eng. J..

[24] Li Y., Yang C., Fang S., Zhou Y., Li M., Liu Z., Zhang X., Duan L., Liu K., Sun F. (2024). Clickable, Thermally Responsive Hydrogels Enabled by Recombinant Spider Silk Protein and Spy Chemistry for Sustained Neurotrophin Delivery. Adv. Mater..

[25] Ma C., Shi Y., Pena D.A., Peng L., Yu G. (2015). Thermally Responsive Hydrogel Blends: A General Drug Carrier Model for Controlled Drug Release. Angew. Chem. Int. Ed..

[26] Kim T.H., Choi J.G., Byun J.Y., Jang Y., Kim S.M., Spinks G.M., Kim S.J. (2019). Biomimetic Thermal-sensitive Multi-transform Actuator. Sci. Rep..

[27] Zhang J.-T., Jandt K.D. (2008). A Novel Approach to Prepare Porous Poly(N-isopropylacrylamide) Hydrogel with Superfast Shrinking Kinetics. Macromol. Rapid Commun..

[28] Luo R., Wu J., Dinh N.-D., Chen C.-H. (2015). Gradient Porous Elastic Hydrogels with Shape-Memory Property and Anisotropic Responses for Programmable Locomotion. Adv. Funct. Mater..

[29] Warren H., Shepherd D.J., In Het Panhuis M., Officer D.L., Spinks G.M. (2020). Porous PNIPAm hydrogels: Overcoming diffusion-governed hydrogel actuation. Sens. Actuators A Phys..

[30] Liu W., Wang Z., Serna J.A., Debastiani R., Gomez J.E.U., Lu L., Yang W., Dong Z., Levkin P.A. (2024). Enhancing Temperature Responsiveness of PNIPAM Through 3D-Printed Hierarchical Porosity. Adv. Funct. Mater..

[31] Zhang J.-T., Huang S.-W., Xue Y.-N., Zhuo R.-X. (2005). Poly(N-isopropylacrylamide) Nanoparticle-Incorporated PNIPAAm Hydrogels with Fast Shrinking Kinetics. Macromol. Rapid Commun..

[32] Xia L.-W., Xie R., Ju X.-J., Wang W., Chen Q., Chu L.-Y. (2013). Nano-structured smart hydrogels with rapid response and high elasticity. Nat. Commun..

[33] Cho Y.E., Park J.-M., Song W.J., Lee M.-G., Sun J.-Y. (2024). Solvent Engineering of Thermo-Responsive Hydrogels Facilitates Strong and Large Contractile Actuations. Adv. Mater..

[34] Ma Y., Lu Y., Yue Y., He S., Jiang S., Mei C., Xu X., Wu Q., Xiao H., Han J. (2024). Nanocellulose-mediated bilayer hydrogel actuators with thermo-responsive, shape memory and self-sensing performances. Carbohydr. Polym..

[35] Bai L., Jin Y., Shang X., Jin H., Zeng W., Shi L. (2023). Dual thermo-responsive multifunctional ionic conductive hydrogel by salt modulation strategy for multilevel encryption and visual monitoring. Chem. Eng. J..

[36] Dixit A., Bag D.S. (2022). Highly stretchable and tough thermo-responsive double network (DN) hydrogels: Composed of PVA-borax and poly (AM-co-NIPAM) polymer networks. Eur. Polym. J..

[37] Bauman L., Zhao B. (2023). Multi-thermo responsive double network composite hydrogel for 3D printing medical hydrogel mask. J. Colloid Interface Sci..

[38] Liu H., Jia X., Liu R., Chen K., Wang Z., Lyu T., Cui X., Zhao Y., Tian Y. (2022). Multifunctional gradient hydrogel with ultrafast thermo-responsive actuation and ultrahigh conductivity. J. Mater. Chem. A.

[39] Zhu P., Deng Y., Wang C. (2017). Graphene/cyclodextrin-based nanocomposite hydrogel with enhanced strength and thermo-responsive ability. Carbohydr. Polym..

[40] Xiu H., Zhao H., Dai L., Li J., Wang Z., Cui Y., Bai Y., Zheng X., Li J. (2022). Robust and adhesive lignin hybrid hydrogel as an ultrasensitive sensor. Int. J. Biol. Macromol..

[41] Abolore R.S., Jaiswal S., Jaiswal A.K. (2025). A comprehensive review on sustainable lignin extraction techniques, modifications, and emerging applications. Ind. Crops Prod..

[42] Pan X., Pan J., Li X., Wang Z., Ni Y., Wang Q. (2024). Tough Supramolecular Hydrogels Crafted via Lignin-Induced Self-Assembly. Adv. Mater..

[43] Li X., You X., Wang X., Kang J., Zhang H.J. (2025). Advanced Lignin-Based Hydrogels with Superior Stiffness, Toughness, and Sensing Capabilities. Adv. Funct. Mater..

[44] You X., Wang X., Zhang H.J., Cui K., Zhang A., Wang L., Yadav C., Li X. (2020). Supertough Lignin Hydrogels with Multienergy Dissipative Structures and Ultrahigh Antioxidative Activities. ACS Appl. Mater. Interfaces.

[45] Arsuffi B., Magrini T., Champeau M., Siqueira G., Titotto S. (2025). 4D printing of natural materials: A review. Sustain. Mater. Technol..

[46] Zhang X.-Z., Xu X.-D., Cheng S.-X., Zhuo R.-X. (2008). Strategies to improve the response rate of thermosensitive PNIPAAm hydrogels. Soft Matter.

[47] Liu K., Cao H., Yuan W., Bao Y., Shan G., Wu Z.L., Pan P. (2020). Stereocomplexed and homocrystalline thermo-responsive physical hydrogels with a tunable network structure and thermo-responsiveness. J. Mater. Chem. B.

[48] Li S., Wang W., Li W., Xie M., Deng C., Sun X., Wang C., Liu Y., Shi G., Xu Y. (2021). Fabrication of Thermoresponsive Hydrogel Scaffolds with Engineered Microscale Vasculatures. Adv. Funct. Mater..

[49] Yan Q., Ding R., Zheng H., Li P., Liu Z., Chen Z., Xiong J., Xue F., Zhao X., Peng Q. (2023). Bio-Inspired Stimuli-Responsive Ti_3_C_2_T_x_/PNIPAM Anisotropic Hydrogels for High-Performance Actuators. Adv. Funct. Mater..

[50] Zhuo R.-X., Li W. (2003). Preparation and characterization of macroporous poly(N-isopropylacrylamide) hydrogels for the controlled release of proteins. J. Polym. Sci. Part A Polym. Chem..

[51] Teubner M., Strey R. (1987). Origin of the scattering peak in microemulsions. J. Chem. Phys..

[52] Guo H., Mussault C., Brûlet A., Marcellan A., Hourdet D., Sanson N. (2016). Thermoresponsive Toughening in LCST-Type Hydrogels with Opposite Topology: From Structure to Fracture Properties. Macromolecules.

[53] Cui K., Sun T., Liang X., Nakajima K., Ye Y., Chen L., Kurokawa T., Gong J. (2018). Multiscale Energy Dissipation Mechanism in Tough and Self-Healing Hydrogels. Phys. Rev. Lett..

[54] Huang Y., Xiao L., Zhou J., Liu T., Yan Y., Long S., Li X. (2021). Strong Tough Polyampholyte Hydrogels via the Synergistic Effect of Ionic and Metal–Ligand Bonds. Adv. Funct. Mater..

[55] Sun T.L., Luo F., Kurokawa T., Karobi S.N., Nakajima T., Gong J.P. (2015). Molecular structure of self-healing polyampholyte hydrogels analyzed from tensile behaviors. Soft Matter.

[56] Lin W.-C., Fan W., Marcellan A., Hourdet D., Creton C. (2010). Large Strain and Fracture Properties of Poly(dimethylacrylamide)/Silica Hybrid Hydrogels. Macromolecules.

[57] Wang Z., Zhou H., Chen W., Li Q., Yan B., Jin X., Ma A., Liu H., Zhao W. (2018). Dually Synergetic Network Hydrogels with Integrated Mechanical Stretchability, Thermal Responsiveness, and Electrical Conductivity for Strain Sensors and Temperature Alertors. ACS Appl. Mater. Interfaces.

[58] Wang H.-X., Zhao X.-Y., Jiang J.-Q., Liu Z.-T., Liu Z.-W., Li G. (2022). Thermal-Responsive Hydrogel Actuators with Photo-Programmable Shapes and Actuating Trajectories. ACS Appl. Mater. Interfaces.

[59] Li J., Ma Q., Xu Y., Yang M., Wu Q., Wang F., Sun P. (2020). Highly Bidirectional Bendable Actuator Engineered by LCST–UCST Bilayer Hydrogel with Enhanced Interface. ACS Appl. Mater. Interfaces.

[60] Fan Z., Xu W., Wang R., Wu H., Liu A. (2023). Fast-response thermo-sensitive actuator based on asymmetric structured PNIPAM hydrogel with inorganic particles embedding. Macromol. Res..

[61] Wang Y., Song C., Yu X., Liu L., Han Y., Chen J., Fu J. (2017). Thermo-responsive hydrogels with tunable transition temperature crosslinked by multifunctional graphene oxide nanosheets. Compos. Sci. Technol..

[62] Kuroki S., Kubota M., Haraguchi R., Oishi Y., Narita T. (2023). Additive-Free Method for Enhancing the Volume Phase Transition Rate in Light-Responsive Hydrogels: A Study of Micro-Nano Bubble Water on PNIPAM-co-AAc Hydrogels. Gels.

[63] Yang T., Wang M., Jia F., Ren X., Gao G. (2020). Thermo-responsive shape memory sensors based on tough, remolding and anti-freezing hydrogels. J. Mater. Chem. C.

[64] Zhao L., Huang J., Zhang Y., Wang T., Sun W., Tong Z. (2017). Programmable and Bidirectional Bending of Soft Actuators Based on Janus Structure with Sticky Tough PAA-Clay Hydrogel. ACS Appl. Mater. Interfaces.

[65] Zhang M., Yang Y., Li M., Shang Q., Xie R., Yu J., Shen K., Zhang Y., Cheng Y. (2023). Toughening Double-Network Hydrogels by Polyelectrolytes. Adv. Mater..

[66] Cui K., Sun T.L., Kurokawa T., Nakajima T., Nonoyama T., Chen L., Gong J.P. (2016). Stretching-induced ion complexation in physical polyampholyte hydrogels. Soft Matter.

[67] Li Z., Zheng Z., Yang Y., Fang G., Yao J., Shao Z., Chen X. (2016). Robust Protein Hydrogels from Silkworm Silk. ACS Sustain. Chem. Eng..

[68] Zhao Y., Nakajima T., Yang J.J., Kurokawa T., Liu J., Lu J., Mizumoto S., Sugahara K., Kitamura N., Yasuda K. (2014). Proteoglycans and Glycosaminoglycans Improve Toughness of Biocompatible Double Network Hydrogels. Adv. Mater..

[69] Nakajima T., Sato H., Zhao Y., Kawahara S., Kurokawa T., Sugahara K., Gong J.P. (2012). A Universal Molecular Stent Method to Toughen any Hydrogels Based on Double Network Concept. Adv. Funct. Mater..

[70] Zhou Y., Kosugi K., Yamamoto Y., Kawahara S. (2017). Effect of non-rubber components on the mechanical properties of natural rubber. Polym. Adv. Technol..

[71] He L., Vibhagool S., Zhao H., Hoven V., Theato P. (2018). Photocaged PNIPAM: A Light Tunable Thermal Responsive Polymer. Macromol. Chem. Phys..

[72] Kuenstler A.S., Lahikainen M., Zhou H., Xu W., Priimagi A., Hayward R.C. (2020). Reconfiguring Gaussian Curvature of Hydrogel Sheets with Photoswitchable Host–Guest Interactions. ACS Macro Lett..

[73] Wang E., Desai M.S., Lee S.-W. (2013). Light-Controlled Graphene-Elastin Composite Hydrogel Actuators. Nano Lett..

[74] Osada Y., Okuzaki H., Hori H. (1992). A Polymer Gel with Electrically Driven Motility. Nature.

[75] Song P.A., Zhang Y., Kuang J. (2007). Preparation and Characterization of Hydrophobically Modified Polyacrylamide Hydrogels by Grafting Glycidyl Methacrylate. J. Mater. Sci..

[76] Takashima Y., Hatanaka S., Otsubo M., Nakahata M., Kakuta T., Hashidzume A., Yamaguchi H., Harada A. (2012). Expansion–Contraction of Photoresponsive Artificial Muscle Regulated by Host–Guest Interactions. Nat. Commun..

[77] Ma Y., Hua M., Wu S., Du Y., Pei X., Zhu X., Zhou F., He X. (2020). Bioinspired High-Power-Density Strong Contractile Hydrogel by Programmable Elastic Recoil. Sci. Adv..

[78] Dong L., Agarwal A.K., Beebe D.J., Jiang H. (2006). Adaptive Liquid Microlenses Activated by Stimuli-Responsive Hydrogels. Nature.

[79] Gladman A.S., Matsumoto E.A., Nuzzo R.G., Mahadevan L., Lewis J.A. (2016). Biomimetic 4D printing. Nat. Mater..

[80] Palleau E., Morales D., Dickey M.D., Velev O.D. (2013). Reversible Patterning and Actuation of Hydrogels by Electrically Assisted Ionoprinting. Nat. Commun..

[81] Lv Z., Xu J., Li C., Dai L., Li H., Zhong Y., Si C. (2021). pH-Responsive Lignin Hydrogel for Lignin Fractionation. ACS Sustain. Chem. Eng..

[82] Parvathy P., Ayobami A.V., Raichur A.M., Sahoo S.K. (2021). Methacrylated Alkali Lignin Grafted P(Nipam-Co-AAc) Copolymeric Hydrogels: Tuning the Mechanical and Stimuli-Responsive Properties. Int. J. Biol. Macromol..

[83] Sun L., Mo Z., Li Q., Zheng D., Qiu X., Pan X. (2021). Facile Synthesis and Performance of pH/Temperature Dual-Response Hydrogel Containing Lignin-Based Carbon Dots. Int. J. Biol. Macromol..

[84] Chandna S., Thakur N.S., Kaur R., Bhaumik J. (2020). Lignin–Bimetallic Nanoconjugate Doped pH-Responsive Hydrogels for Laser-Assisted Antimicrobial Photodynamic Therapy. Biomacromolecules.

[85] Dai L., Ma M., Xu J., Si C., Wang X., Liu Z., Ni Y. (2020). All-Lignin-Based Hydrogel with Fast pH-Stimuli Responsiveness for Mechanical Switching and Actuation. Chem. Mater..

[86] Liu W., Ye Z., Liu D., Wu Z. (2018). Hydrogels Derived from Lignin with pH Responsive and Magnetic Properties. BioResources.

