# Supplementary material for: Strong, Fast-Response Printable Lignin/PNIPAM Thermo-Responsive Hydrogel via Hierarchical Phase Separation
Source: Gels. 2026 Apr 27;12(5):362. doi: 10.3390/gels12050362 (PMC13206440; doi:10.3390/gels12050362)
Supplement: Supplementary file 1 [file gels-12-00362-s001.zip › gels-4277372-Supporting information.pdf]

Supporting information

Strong, Fast-Response Printable Lignin/PNIPAM Ther-mo-Responsive Hydrogel via Hierarchical Phase Separation

Qian Wang, Hui Jie Zhang, Wenlong Zhang, Linbin Li, Yifan Zhang, Ping Rao*, and Xiangyu You*

Qian Wang and Hui Jie Zhang should be considered joint first author.

Q. Wang, H. J. Zhang, W. Zhang, L. Li, X. You
College of Bioresources Chemical and Materials Engineering, Shaanxi University of Science &Technology, Xi'an, Shaanxi, 710021, China
E-mail: hjzhang@sust.edu.cn

Y. Zhang
Department of Engineering Mechanics, Zhejiang University, Hangzhou, Zhejiang 310027, China

P. Rao
State Key Laboratory of Fluid Power & Mechatronic System, Key Laboratory of Soft Machines and Smart Devices of Zhejiang Province, Center for X-Mechanics, and Department of Engineering Mechanics, Zhejiang University, Hangzhou, Zhejiang 310027, China
E-mail: raoping@zju.edu.cn

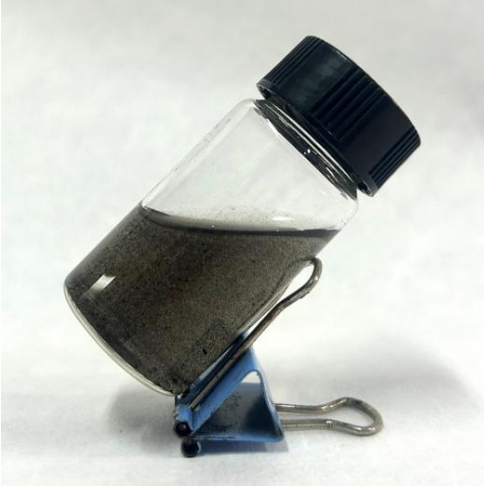

Figure S1. Aqueous Solution of PNIPAM/lignin.

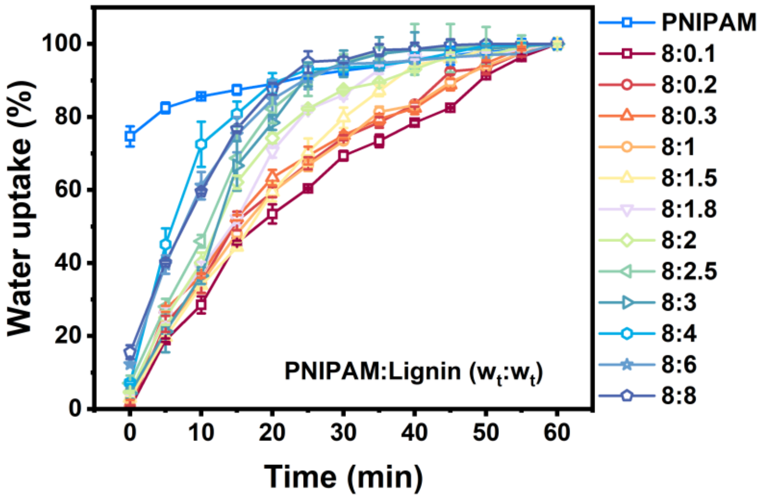

Figure S2. Water uptake rate of the heating-induced shrunk NL hydrogels.

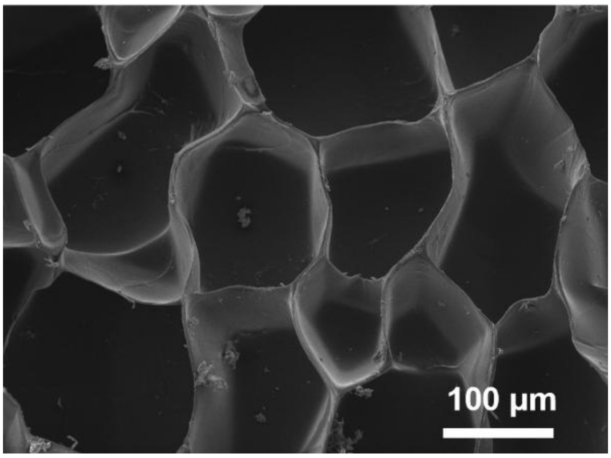

Figure S3. The SEM image of the cross-section of the PNIPAM hydrogel.

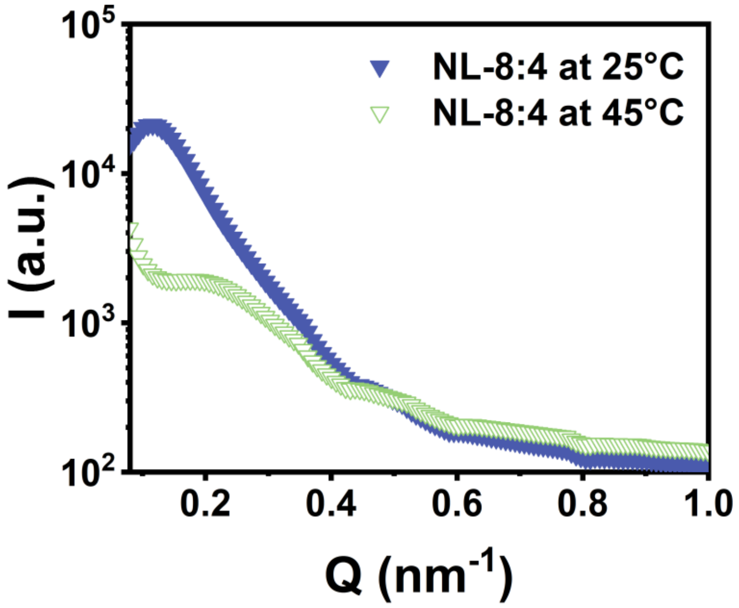

Figure S4. The 1D SAXS profile of the NL-8:4 gel at 25°C and 45°C.

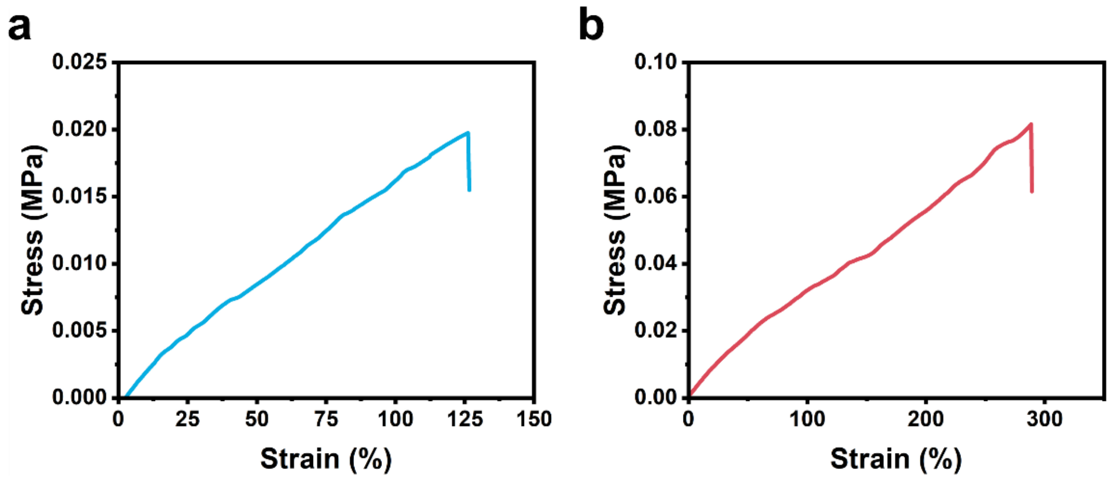

Figure S5. Tensile stress-strain curves of chemically crosslinked PNIPAM hydrogels: (a) at 25°C and (b) at 45°C.

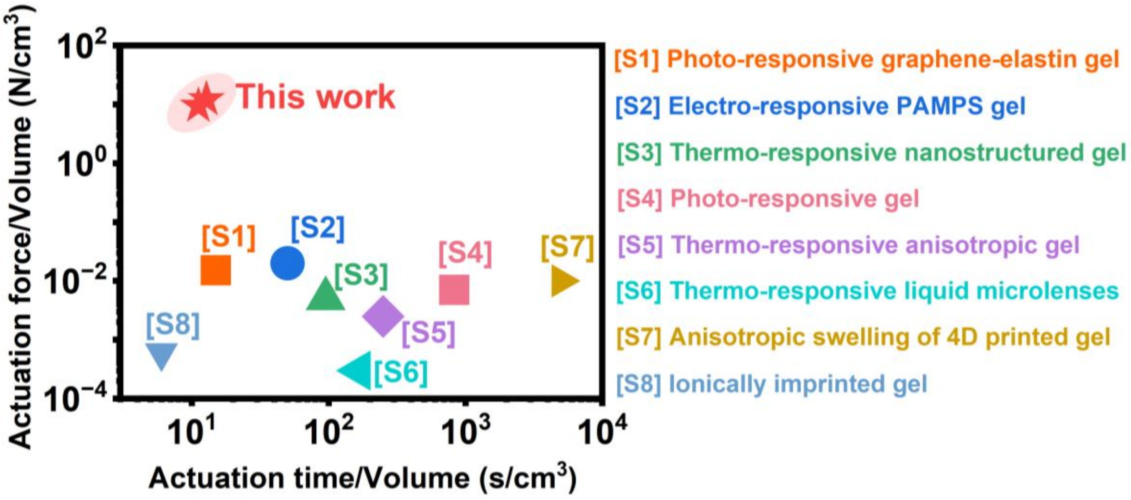

Figure S6. Comparison in the Actuation performance of NL hydrogel with other responsive hydrogel actuators[S1-S8].

Table S1. Comparison of Composition and Properties of NL Hydrogels and Lignin-Based Stimuli-Responsive Hydrogels

Lignin Type/Source	Lignin Content	Responsiveness	Response Time	Mechanical Properties	Application	Preparation Method	Ref.
Acetic acid lignin (bamboo residue)	0.3-21.2 wt% lignin/DMF solution	Thermal response	10 s to 90% water expulsion	below LCST: >1 MPa stress, 500% strain; above LCST: ~5 MPa stress, 1500% strain	Actuators, intelligent manipulators	Drying-swelling process	This work
Alkaline lignin (corn cob)	Main network component	pH response	16 min to >180% water content	N/A	Lignin fractionation	Chemical crosslinking	[S9]
Alkali lignin (softwood/hardwood)	LMA content: 4.5-16 wt%	pH/thermal dual response	75 min to deswelling equilibrium (temp.)	0.12 MPa stress, 24% strain	Tissue engineering scaffolds; wound healing	Lignin methacrylation + free radical polymerization	[S10]
p-Toluenesulfonic acid lignin (corn cob)	Used to prepare L-CDs; 0.5 mL L-CDs solution per hydrogel batch	pH/thermal dual response	5 min to deswelling (temp.)	N/A	Tissue engineering scaffolds; drug carriers	Free radical polymerization	[S11]
Kraft lignin (agricultural biomass)	Used to synthesize lignin-metallic/bimetallic nanocomplexes (LNCs)	pH response	360 min to complete degradation (pH 5.5)	N/A	Wound healing; antimicrobial nanocoatings	Nanocomplexes synthesis + hydrogel doping	[S12]
Kraft lignin (hardwood black liquor)	Main network component	pH response	1 min per bending cycle (pH 2/10)	0.036 MPa stress, 29.4% strain	Hydrogel actuators	One-step chemical crosslinking	[S13]
Alkaline lignin (byproduct of hardwood alkaline pulping)	0.5 g per formulation	pH/magnetic dual response	13.5 min to 100% swelling (pH 6.86)	N/A	Drug release, heavy metal adsorbents	Free radical polymerization	[S14]

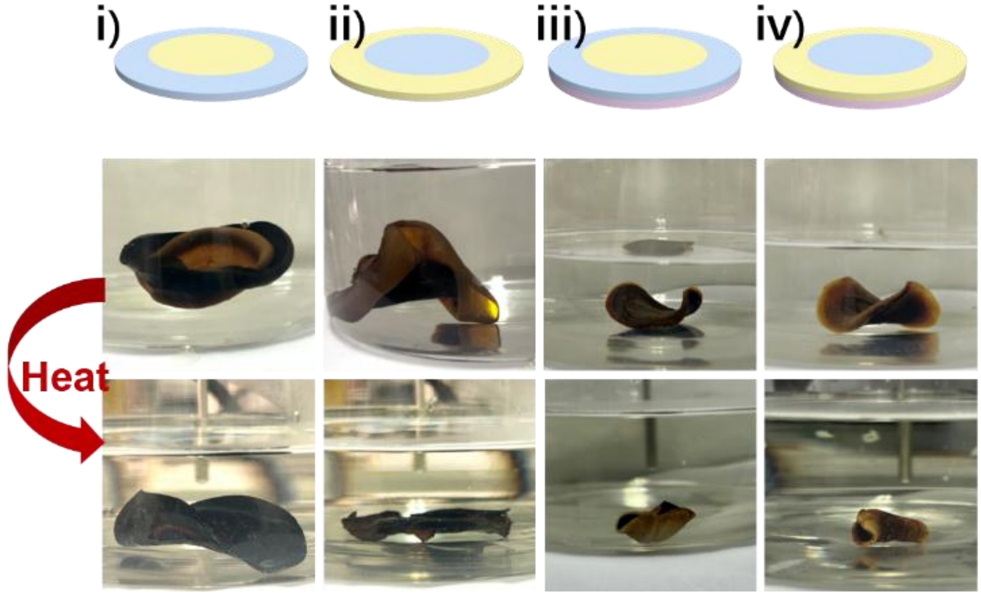

Figure S7. Shape deformation of the NL hydrogel disk printed with different NL hydrogels: a) the schematic illustration of the composition of the hydrogel disks (yellow for NL-8:1.5 gel, blue for NL-8:6 gel, and pink for PNIPAM gel); b) the photos of printed hydrogels at the swollen state in 25°C water; c) the photos of the hydrogels after being heated to 45°C.

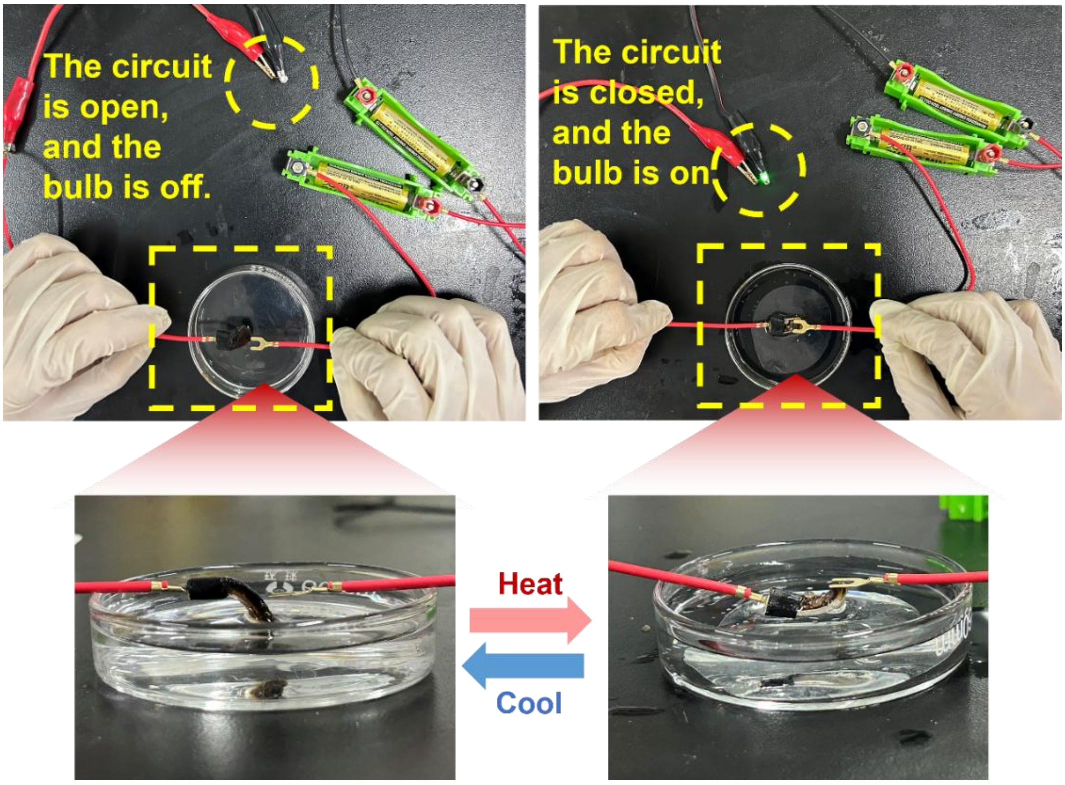

Figure S8. Soft switching of NL/PNIPAM bilayer hydrogel actuators controlled by temperature.

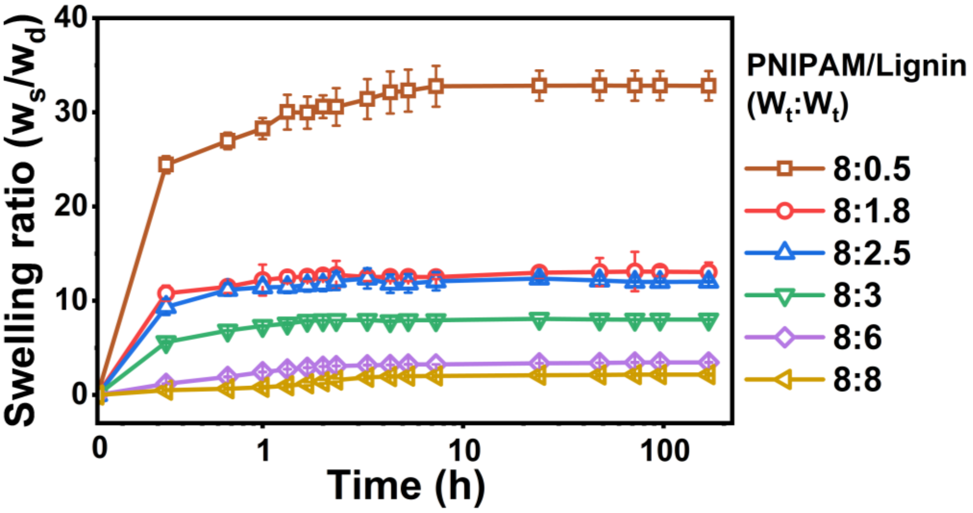

Figure S9. Swelling equilibrium curve of NL hydrogels (w_d is the dry weight of the hydrogel, and w_s is calculated as the weight of the equilibrium-swollen hydrogel at regular time intervals minus w_d).

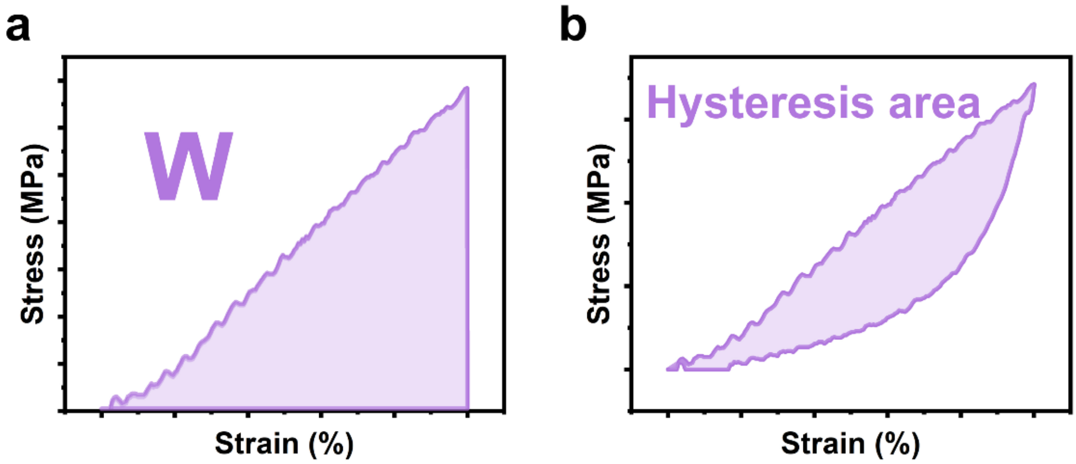

Figure S10. Schematic diagram illustrating the calculation of (a) work of extension at fracture (W) and (b) hysteresis area.

Reference:

[S1] Wang E, Desai M S, Lee S-W., 2013. Light-Controlled Graphene-Elastin Composite Hydrogel Actuators. Nano Letters. 13, 2826-30.

[S2] Osada Y, Okuzaki H, Hori H., 1992. A Polymer Gel with Electrically Driven Motility. Nature. 355, 242-4.

[S3] Song P A, Zhang Y, Kuang J.,2007. Preparation and Characterization of Hydrophobically Modified Polyacrylamide Hydrogels by Grafting Glycidyl Methacrylate. Journal of Materials Science. 42, 2775-81.

[S4] Takashima Y, Hatanaka S, Otsubo M, et al., 2012. Expansion–Contraction of Photoresponsive Artificial Muscle Regulated by Host–Guest Interactions. Nature Communications, 3, 1270.

[S5] Ma Y, Hua M, Wu S, et al., 2020. Bioinspired High-Power-Density Strong Contractile Hydrogel by Programmable Elastic Recoil. Science Advances. 6, eabd2520.

[S6] Dong L, Agarwal A K, Beebe D J, et al., 2006. Adaptive Liquid Microlenses Activated by Stimuli-Responsive Hydrogels. Nature. 442, 551-4.

[S7] Sydney Gladman A, Matsumoto E A, Nuzzo R G, et al., 2016. Biomimetic 4D printing. Nature Materials. 15, 413-8.

[S8] Palleau E, Morales D, Dickey M D, et al., 2013. Reversible Patterning and Actuation of Hydrogels by Electrically Assisted Ionoprinting. Nature Communications. 4, 2257.

[S9] Lv, Z., Xu, J., Li, C., Dai, L., Li, H., Zhong, Y., et al., 2021. pH-Responsive Lignin Hydrogel for Lignin Fractionation. ACS Sustainable Chemistry & Engineering. 9, 13972-13978.

[S10] Parvathy, P.A., Ayobami, A.V., Raichur, A.M. and Sahoo, S.K., 2021. Methacrylated Alkali Lignin Grafted P(NIPAM-Co-AAc) Copolymeric Hydrogels: Tuning the Mechanical and Stimuli-Responsive Properties. International Journal of Biological Macromolecules. 192, 180-196.

[S11] Sun, L., Mo, Z., Li, Q., Zheng, D., Qiu, X. and Pan, X., 2021. Facile Synthesis and Performance of pH/Temperature Dual-Response Hydrogel Containing Lignin-Based Carbon Dots. International Journal of Biological Macromolecules. 175, 516-525.

[S12] Chandna, S., Thakur, N.S., Kaur, R. and Bhaumik, J., 2020. Lignin–Bimetallic Nanoconjugate Doped pH-Responsive Hydrogels for Laser-Assisted Antimicrobial

Photodynamic Therapy. Biomacromolecules. 21, 3216-3230.
[S13] Dai, L., Ma, M., Xu, J., Si, C., Wang, X., Liu, Z., et al., 2020. All-Lignin-Based Hydrogel with Fast pH-Stimuli Responsiveness for Mechanical Switching and Actuation. Chemistry of Materials. 32, 4324-4330.
[S14] Liu, W., Ye, Z., Liu, D., and Wu, Z., 2018. Hydrogels Derived from Lignin with pH Responsive and Magnetic Properties. Bioresources. 13, 7281-7293.

.

Supporting movies:

Movie S1. The hydrogel patterns were fabricated via direct ink writing (DIW) technology, dried, and then immersed in water.

Movie S2. The reverse bending process of the bilayer NL hydrogel strips prepared from NL-8:2 and NL-8:5 in water at 45°C.

Movie S3. The bilayer hydrogel gripper successfully captured the silicone block in water at 45°C.

Movie S4. The response process of NL-8:0.2 and PNIPAM hydrogel discs to NIR irradiation.
